# Authenticity Assessment of Five Monofloral Honeys Based on Phytochemical Profiles

**DOI:** 10.3390/foods15111954

**Published:** 2026-06-01

**Authors:** Yinan Du, Xinyue Du, Yue Wang, Hongcheng Zhang, Jiangtao Qiao, Yuncai Lu

**Affiliations:** 1 College of Life Sciences and Food Engineering, Hebei University of Engineering, Handan 056038, China; duyinan2026@163.com; 2 College of Food Engineering, Harbin University of Commerce, Harbin 150023, China; 3 College of Advanced Agriculture and Ecological Environment, Heilongjiang University, Harbin 150080, China; 4 State Key Laboratory of Resource Insects, Institute of Apicultural Research, Chinese Academy of Agricultural Sciences, Beijing 100093, China; zhanghongcheng@caas.cn

**Keywords:** fruit monofloral honeys, phytochemicals, characteristic components, HPLC fingerprints, authenticity evaluation

## Abstract

Background: Ensuring authenticity and verifying the floral origin of honey are persistent and critical issues in the quality control of bee products; in particular, the characteristic components and practical authenticity evaluation standards of several specialty fruit monofloral honeys are still insufficiently defined. Methods: To address this, we conducted a comparative analysis of five fruit monofloral honey (loquat, pomegranate, citrus, apple, and blueberry) phytochemicals using high-performance liquid chromatography (HPLC), liquid chromatography–mass spectrometry (LC-MS), and the Similarity Evaluation System for Chromatographic Fingerprint of Traditional Chinese Medicine (TCM). Results: Based on the currently available literature and databases, eleven identified phytochemicals appear to be reported in honey for the first time, including quinic acid derivatives, phenolamides, and flavonoid glycosides. Characteristic components with high species dependence were identified in distinct honey samples: anisic acid in loquat honey; methyl syringate in pomegranate honey; caffeine in citrus honey; cinnamic acid and methyl syringate in apple honey; and phaseic acid, methyl syringate, isorhamnetin-3-O-neohesperidoside, and callunene in blueberry honey. Twenty-three commercial samples were collected from the retail market to assess authenticity using HPLC fingerprints and quantitative thresholds for characteristic components. Authenticity was assessed based on both chromatographic fingerprint similarity and the content thresholds of characteristic phytochemicals specific to each monofloral honey type. The results indicated that 19 commercial samples satisfied the proposed authenticity criteria, whereas four commercial samples showed inconsistencies in characteristic phytochemical profiles or fingerprint similarity. Conclusions: This research establishes reliable chemical markers and a quantitative method to assess the authentication of five monofloral honeys, supporting high-value product development.

## 1. Introduction

Honey, as a naturally occurring sweetener produced by honeybees from floral nectar and mixed with secretions, has long been appreciated for its nutritional and therapeutic value [1]. Honey is a complex matrix that contains not only carbohydrates but also numerous minor components, including amino acids, enzymes, organic acids, minerals, vitamins, polyphenols, flavonoids, and terpenoids [2]. These phytochemicals are recognized as the major contributors to the biological activity of honey, which includes antioxidant [3], antimicrobial [4], anti-inflammatory [5], and wound-healing properties [6]. Due to such nutritional and health-promoting functions, the global demand for honey has been steadily increasing. However, the growing market demand has simultaneously increased the incidence of honey adulteration and mislabeling. Honey is currently regarded as one of the most vulnerable food commodities to fraudulent practices worldwide, following milk and olive oil [7]. Such adulteration not only undermines consumer trust and market stability but also threatens the viability of beekeeping and ecological sustainability. Therefore, developing reliable methods to evaluate honey authenticity has become a formidable scientific and practical challenge.

Traditional methods for identifying honey authenticity mainly rely on sensory evaluation combined with melissopalynological (pollen) examination [8]. However, due to natural variations in pollen content, this approach may lead to misjudgment. The other common methods for performing honey authentication rely on physicochemical parameters [9]. Although effective in some cases, these approaches are not sufficient to detect all forms of adulteration, especially when the adulterants closely mimic the natural sugar profile of honey [10]. Therefore, combining these traditional techniques with modern analytical methods can effectively improve the accuracy of honey authenticity determination. At present, honey adulteration is commonly achieved by adding cane syrup, corn syrup, maltose syrup, invert syrup, rice syrup, beet syrup, or cassava syrup [11]. Given the differences in carbon isotope proportions between C3 and C4 plants, the stable carbon isotope proportion method has been extensively applied to distinguish syrup adulteration [12]. However, this method can only identify adulteration with corn and cane syrups, and it is ineffective for adulteration with C3 plant syrups derived from rice, wheat, or beet [1]. Recently, the scientific focus has shifted toward the intrinsic phytochemicals that nectariferous plants transfer into honey via bee-collected nectar. These inherent compounds, including phenolic acids, flavonoids, and terpenoids, are highly species-dependent and vary considerably among plant species, thus serving as reliable markers for verifying botanical origins and authenticity. For instance, methyl syringate has been recognized as a distinct marker in rapeseed honey, phaseic acid in acacia honey, and lindenin in linden honey [13]. Similarly, unique terpenoids such as abscisic acids, callunene, and schefflerin have been identified in longan, litchi, and schefflera honeys, respectively [14]. These studies demonstrate the considerable value of intrinsic phytochemicals in the differentiation and authentication of monofloral honeys. Moreover, chemometric approaches combined with chromatographic and metabolomic analyses, including principal component analysis (PCA), partial least squares discriminant analysis (PLS-DA), and orthogonal partial least squares discriminant analysis (OPLS-DA), have been increasingly applied in honey authenticity and botanical origin studies. These multivariate statistical methods can effectively reveal compositional differences among honey samples and improve the classification performance [15].

Despite these advances, numerous studies have largely concentrated on widely consumed honeys and high-yield monofloral honeys such as acacia, linden, rapeseed, longan, litchi, and jujube, among others [8]. In contrast, monofloral honeys, although increasingly popular among consumers, remain comparatively underexplored. Among monofloral honeys, loquat (*Eriobotrya japonica*) honey is known for its unique flavor and cough-relieving properties. Previous studies have shown that hydroxybenzoic acid and protocatechuic aldehyde can serve as characteristic floral markers of this honey [16]. Pomegranate (*Punica granatum*) honey is rich in phenolic acids and flavonoids, including gallic acid, caffeic acid, quercetin, apigenin, luteolin, sakuranetin, and pinocembrin [17]. Apple (*Malus domestica*) honey, although less frequently reported, is expected to exhibit characteristic phytochemical signatures associated with apple flowers. Citrus (*Citrus* spp.) honey is among the most commercially important monofloral honeys worldwide, characterized by abundant phenolic acids and flavonoids, such as caffeic acid, *p*-coumaric acid, and hesperetin [18]. Blueberry (*Vaccinium* spp.) honey is relatively rare and considered chemically distinctive; however, current knowledge regarding its phytochemical composition remains limited, and clear characteristic marker compounds have not yet been established. More importantly, there is still a lack of a systematic and practically applicable frameworks for simultaneously differentiating fruit monofloral honeys and evaluating the authenticity of commercial samples.

Therefore, this study aimed to characterize the phytochemical profiles of five fruit monofloral honeys, namely loquat, apple, blueberry, pomegranate, and citrus honeys, and to determine the characteristic markers for each of these five monofloral honeys. Based on quantitative thresholds of characteristic markers and fingerprint analysis, an authenticity evaluation method for the five monofloral honeys was developed.

## 2. Materials and Methods

### 2.1. Honey Samples

Raw honey samples were collected from multiple apiaries distributed across major production regions in China. Specifically, 13 loquat honey samples were collected from Fujian, Zhejiang, and Jiangsu Provinces; 6 pomegranate honey samples from Henan, Yunnan, and Xinjiang Provinces; 8 citrus honey samples from Hunan, Hubei, and Sichuan Provinces in 2024; 6 apple honey samples from Shandong and Shaanxi Provinces; and 10 blueberry honey samples from Heilongjiang, Liaoning, and Guizhou Provinces.

To minimize interference due to mixed floral sources, apiary locations and bee foraging areas were selected in regions dominated by corresponding monofloral orchards. The botanical origins of the honey samples were preliminarily determined according to the hive locations and flowering conditions during the nectar collection periods. Melissopalynological analysis was subsequently performed using a phase-contrast microscope, following the method reported by Lutier et al. [19]. Representative pollen micrographs are presented in Supplementary Figure S1, while the pollen analysis results are summarized in Supplementary Table S1.

In addition, 23 commercial honey samples of loquat, pomegranate, apple, citrus, and blueberry honeys were randomly obtained from supermarkets and online retail platforms in China during 2024–2025. Detailed information regarding the manufacturing dates and product origins is provided in Supplementary Table S2. All samples were preserved at −18 °C before analysis.

### 2.2. Chemicals

Standards of chlorogenic acid, 3-*O*-*p*-coumaroylquinic acid, 5-*O*-feruloylquinic acid, anisic acid (4-methoxybenzoic acid), 4-hydroxybenzoic acid, cinnamic acid, methyl syringate, diosmetin, 8-methoxy kaempferol, 5-methoxy pinobanksin, pinocembrin, chrysin, quercetin, luteolin, apigenin, kaempferol, hyperoside (quercetin-3-*O*-galactoside), isorhamnetin-3-*O*-neohesperidoside, kaempferol-3-*O*-sophoroside, *trans*, *trans*-abscisic acid, *cis*, *trans*-abscisic acid, and caffeine were obtained from Sigma-Aldrich Chemical Co. (St. Louis, MO, USA). N1,N10-di-*p*-coumaroyl-N5-caffeoyl spermidine, tri-*p*-coumaroyl spermidine, di-*p*-coumaroyl spermidine, N1-*p*-coumaroyl-N5,N10-di-caffeoyl spermidine, phaseic acid, and callunene were isolated by ourselves using preparative HPLC. Their structures were confirmed by LC-MS/MS and NMR (^1^H and ^13^C NMR) analyses, together with a comparison to published literature data. The purities of these compounds were higher than 97% based on the HPLC analysis.

HPLC-grade methanol was supplied by Thermo Fisher Scientific (Fair Lawn, NJ, USA). Analytical-grade formic acid, together with HPLC/MS-grade acetic acid, was purchased from J.T. Baker (Phillipsburg, NJ, USA). Ultrapure water was generated using a Milli-Q Integral purification system (Millipore, Billerica, MA, USA). Strata-X-A mixed-mode reversed-phase/anion-exchange SPE cartridges (60 mg/mL) were acquired from Phenomenex (Torrance, CA, USA). All remaining chemicals and reagents were provided by Solarbio (Beijing, China).

### 2.3. Pre-Concentration of Phytochemicals in Honey Samples

Phytochemicals from the five monofloral honey samples were enriched using Strata-X-A SPE cartridges following our previously reported procedure, with slight modifications [20]. Before extraction, the cartridges were sequentially conditioned with 3 mL methanol and equilibrated using 3 mL ultrapure water. Honey samples (20 g) were diluted with 80 mL ultrapure water, and the pH was adjusted to 6.5–7.0 using a 5% ammonia solution (*v*/*v*). After centrifugation at 9000× *g* for 15 min to remove insoluble materials, the resulting supernatants were passed through the preconditioned SPE cartridges at a slow flow rate.

The cartridges were subsequently rinsed with 3 mL ultrapure water to eliminate sugars and other weakly retained substances. Target phytochemicals retained on the cartridges were then eluted using 3 mL formic acid/methanol (1:9, *v*/*v*). The eluates were evaporated to dryness at 40 °C under nitrogen flow (NDK-36W, Shanghai Guansen Technology Co., Ltd., Shanghai, China), reconstituted in 2 mL methanol containing 2% acetic acid, and filtered through a 0.22 μm membrane filter (Millipore, Carrigtowhill, Cork, Ireland) before HPLC analysis.

### 2.4. Analyses of Phytochemicals via HPLC-PDA and HPLC-QTOF-MS/MS

The phytochemical analysis of the enriched honey extracts was performed using a Shimadzu HPLC system equipped with a PDA-20A photodiode array detector, SIL autosampler, CTO-10A column oven, and LC-6AD pump (Shimadzu, Tokyo, Japan). Chromatographic separation was achieved on a Gemini C18 reversed-phase column (150 × 4.6 mm, 3 μm; Phenomenex, CA, USA). The mobile phases consisted of aqueous acetic acid (2%, *v*/*v*; solvent A) and methanolic acetic acid (2%, *v*/*v*; solvent B). The flow rate was maintained at 0.7 mL/min, and the column temperature was controlled at 35 °C.

Compound characterization was further conducted using HPLC-QTOF-MS/MS (Agilent 6520, Agilent Technologies, Palo Alto, CA, USA). The mass spectrometer was operated under the following conditions: source voltage 4 kV, capillary voltage 130 V, capillary temperature 350 °C, scanning range m/z 50–800, and collision energy 20 eV. Compound identification was achieved through comparison of the retention behaviors, UV absorption characteristics, and MS/MS fragmentation patterns with those in authentic reference standards or previously published data. Quantitative analysis was performed using external calibration based on chromatographic peak areas monitored at 270 nm [21]. The analytical method was further validated in terms of linearity, limits of detection (LOD), limits of quantification (LOQ), recovery, and precision. Detailed validation data are provided in Supplementary Table S3.

### 2.5. HPLC Purification of Phytochemicals in Five Monofloral Honeys

To isolate and characterize the phytochemicals from the five monofloral honeys, semi-preparative HPLC purification was conducted using a Shimadzu LC-6AD HPLC system coupled with an FRC-10A fraction collector (Shimadzu, Tokyo, Japan). Separation was performed on a Shim-pack PREP-ODS (H) column (250 × 20 mm, 5 μm) maintained at 30 °C. The mobile phase consisted of 2% aqueous acetic acid (solvent A) and 2% acetic acid in methanol (solvent B), delivered at a flow rate of 3.0 mL/min. The injection volume was set at 5 mL, and all solvents and samples were passed through 0.22 μm membrane filters before purification.

Fractions corresponding to target compounds were collected automatically, evaporated under nitrogen to dryness, and preserved at −80 °C in the absence of light until further analysis.

### 2.6. Statistics

All experiments were conducted in triplicate, and the results are presented as the mean ± standard deviation (SD). Statistical analyses were performed using IBM SPSS Statistics 29.0 (IBM Corp., Armonk, NY, USA). Chromatographic fingerprint similarities were evaluated using the Similarity Evaluation System for Chromatographic Fingerprint of TCM (Chinese Pharmacopoeia Committee, version 2004A) and served as an important criterion for sample evaluation and classification. Principal component analysis (PCA) was performed using Metware Cloud to visualize the overall phytochemical differences among the investigated monofloral honey samples.

## 3. Results

### 3.1. Phytochemical Profiles

**Figure 1:**
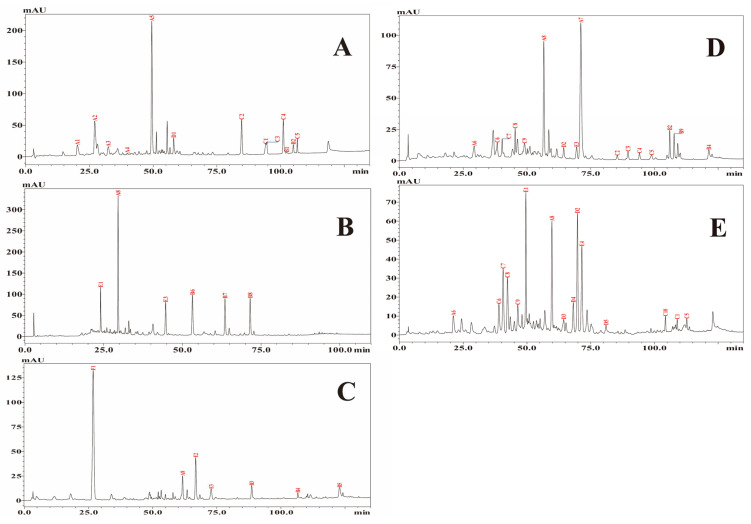
HPLC chromatograms of five monofloral raw honey samples: (**A**) loquat honey; (**B**) pomegranate honey; (**C**) citrus honey; (**D**) apple honey; (**E**) blueberry honey. Notes. A1: chlorogenic acid; A2: isochlorogenic acid; A3: 3-O-p-coumaroylquinic acid; A4: 5-O-feruloylquinic acid; A5: anisic acid; A6: 4-hydroxybenzoic acid; A7: cinnamic acid; A8: methyl syringate; B1: diosmetin; B2: 8-methoxy kaempferol; B3: 5-methoxy pinobanksin; B4: pinocembrin; B5: chrysin; B6: quercetin; B7: luteolin; B8: apigenin; B9: kaempferol; C1: N1,N10-di-*p*-coumaroyl-N5-caffeoyl spermidine; C2: N1(Z),N5(Z),N10(Z)-tri-*p*-coumaroyl spermidine; C3: N1(Z),N5(Z),N10(E)-tri-*p*-coumaroyl spermidine; C4: N1(E),N5(Z),N10(E)-tri-*p*-coumaroyl spermidine; C5: N1(E),N5(E),N10(E)-tri-*p*-coumaroyl spermidine; C6: N1(Z),N10(Z)-di-*p*-coumaroyl spermidine; C7: N1(Z),N10(E)-di-*p*-coumaroyl spermidine; C8: N1(E),N10(Z)-di-*p*-coumaroyl spermidine; C9: N1(E),N10(E)-di-*p*-coumaroyl spermidine; C10: N1-*p*-coumaroyl-N5,N10-di-caffeoyl spermidine; D1: hyperoside (quercetin-3-O-galactoside); D2: isorhamnetin-3-O-neohesperidoside; D3: kaempferol-3-O-sophoroside; D4: kaempferol-3-O-(2″-O-glucosyl)-rutinoside; D5: isorhamnetin-3-O-vicianoside; E1: phaseic acid; E2: *trans*, *trans*-abscisic acid; E3: *cis*, *trans*-abscisic acid; E4: callunene; F1: caffeine.

**Figure 2:**
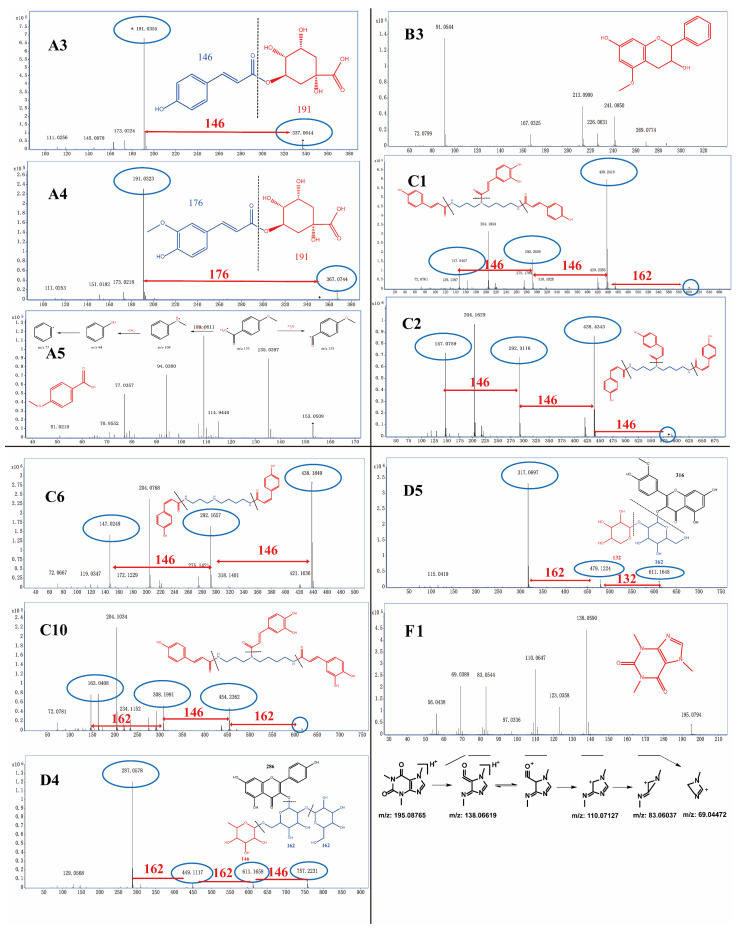
ESI-qTOF-MS/MS spectra and fragment cleavage patterns of characteristic components. Notes. A3: 3-O-p-coumaroylquinic acid; A4: 5-O-feruloylquinic acid; A5: anisic acid; C1: N1,N10-di-*p*-coumaroyl-N5-caffeoyl spermidine; C2: N1(Z),N5(Z),N10(Z)-tri-*p*-coumaroyl spermidine; C6: N1(Z),N10(Z)-di-*p*-coumaroyl spermidine; C10: N1-*p*-coumaroyl-N5,N10-di-caffeoyl spermidine; D4: kaempferol-3-O-(2″-O-glucosyl)-rutinoside; D5: isorhamnetin-3-O-vicianoside; F1: caffeine.

Based on the HPLC profiles of the five monofloral honeys (Figure 1), six classes of compounds were characterized, including 8 phenolic acids and esters, 9 flavonoids, 10 phenolamides, 5 flavonoid glycosides, 4 terpenoids, and 1 alkaloid (Table 1). The MS/MS profiles and cleavage patterns of these compounds are illustrated in Figure 2 and Supplementary Figure S2, respectively.

#### 3.1.1. Phenolic Acids and Esters

Seven phenolic acids and a phenolic acid ester were identified from the five monofloral honeys, namely chlorogenic acid (A1), isochlorogenic acid (A2), 3-O-p-coumaroylquinic acid (A3), 5-O-feruloylquinic acid (A4), anisic acid (A5), 4-hydroxybenzoic acid (A6), cinnamic acid (A7), and methyl syringate (A8). Among them, compounds A1–A5 were identified in loquat honey; compound A6 in apple honey and blueberry honey; compound A7 in apple honey; and compound A8 in apple, blueberry, citrus, and pomegranate honeys.

Compounds A1, A2, A3, and A4 were all identified as quinic acid derivatives. In negative-ion mode, these compounds showed quasi-molecular ions at m/z 353, 353, 337, and 367 [M–H]^−^, respectively (Supplementary Figure S2), and shared a characteristic MS/MS fragment ion at m/z 191, indicating the presence of quinic acid. The neutral losses of 162, 146, and 176 amu corresponded to caffeoyl, p-coumaroyl, and feruloyl residues, respectively. Based on their characteristic fragmentation behaviors, together with comparisons of their retention times, UV λ max values, and MS/MS spectra with commercial standards, the four compounds were unambiguously identified as chlorogenic acid, isochlorogenic acid, 3-O-*p*-coumaroylquinic acid, and 5-O-feruloylquinic acid.

Compound A5 demonstrated a quasi-molecular ion at m/z 153 [M+H]^+^ (Figure 2). It produced fragment ions at m/z 135 [M–H_2_O]^+^ and m/z 109 [M–CO_2_]^+^, indicating the presence of a hydroxyl and a carboxyl group. The fragment ion at m/z 107 further produced a fragment at m/z 77, indicating the loss of methoxyl groups (-OCH_3_). The characteristic fragment ion at m/z 77 indicated the presence of a benzene ring. According to the characteristic fragment ions and cleavage patterns in LC-MS/MS (Figure 2), we identified this component as anisic acid (4-methoxybenzoic acid). This component was further validated by comparing the retention time, UV λ max, and MS/MS spectra with commercial standards. In addition, the MS/MS data also revealed the presence of anisaldehyde; no corresponding anisaldehyde peak was observed in the HPLC chromatogram.

The distribution of phenolic acids varied noticeably among the five fruit monofloral honeys. Loquat honey showed the highest diversity of phenolic acid-related compounds, particularly quinic acid derivatives (A1–A4) and anisic acid (A5), suggesting a relatively richer phenolic acid profile associated with loquat nectar. In contrast, cinnamic acid (A7) was only detected in apple honey, whereas methyl syringate (A8) was widely distributed in apple, blueberry, citrus, and pomegranate honeys, indicating broader occurrence among fruit floral sources. Notably, anisic acid (A5) appeared to exhibit stronger honey-type specificity, as it was exclusively detected in loquat honey. These compositional differences suggest that phenolic acids and their derivatives may contribute substantially to the chemical differentiation of fruit monofloral honeys and could serve as complementary markers for botanical origin discrimination.

#### 3.1.2. Flavonoids

Nine flavonoids were identified, namely diosmetin (B1), 8-methoxy kaempferol (B2), 5-methoxy pinobanksin (B3), pinocembrin (B4), chrysin (B5), quercetin (B6), luteolin (B7), apigenin (B8), and kaempferol (B9). Compound B1 was identified in loquat honey; compound B2 in apple honey and loquat honey; compounds B3 and B4 in citrus honey; compound B5 in apple honey and citrus honey; compounds B6–B8 in loquat honey; and compound B9 in apple honey.

These compounds can be classified into four subclasses: flavones (B1; B5; B7; B8), flavanones (B4), flavonols (B2; B6; B9), and dihydroflavonols (B3). The nine flavonoids (B1–B9) exhibited highly consistent MS/MS fragmentation behaviors characteristic of the flavonoid skeleton (Supplementary Figure S2). A dominant and ubiquitous product ion at m/z 153 was observed in all compounds (Supplementary Figure S2), corresponding to the 1,3A^+^ fragment generated from the Retro-Diels–Alder (RDA) cleavage of the A-ring, which represents the most characteristic fragment ion for flavonoid identification in positive-ion mode [22]. Moreover, the flavonols (B6 and B9) displayed a distinct m/z 137 ion (Supplementary Figure S2), corresponding to B-ring RDA cleavage, which clearly differentiates flavonols from flavones. The dihydroflavonols (B3) showed a different fragmentation pattern from the unsaturated flavones and flavonols. The presence of a saturated C2–C3 bond facilitated side-chain cleavage, generating characteristic ions at m/z 167 (Supplementary Figure S2).

Noticeable differences were also observed in the flavonoid composition among the five fruit monofloral honeys. Loquat honey contained the highest diversity of flavonoids, including flavones and flavonols such as diosmetin (B1), quercetin (B6), luteolin (B7), and apigenin (B8), suggesting a relatively complex flavonoid profile associated with loquat floral origin. In contrast, citrus honey was mainly characterized by flavanone-related compounds, including pinocembrin (B4) and 5-methoxy pinobanksin (B3), whereas apple honey contained characteristic flavonols such as 8-methoxy kaempferol (B2) and kaempferol (B9). These differences in flavonoid subclass distribution may reflect variations in secondary metabolic pathways among different fruit plants. Furthermore, several flavonoids exhibited relatively selective occurrence patterns among honey types, indicating their potential usefulness as complementary markers for distinguishing fruit monofloral honeys and evaluating their botanical origins.

#### 3.1.3. Phenolamides

Ten phenolamides were identified as follows: N1,N10-di-*p*-coumaroyl-N5-caffeoyl spermidine (C1); N1(Z),N5(Z),N10(Z)-tri-*p*-coumaroyl spermidine (C2); N1(Z),N5(Z),N10(E)-tri-*p*-coumaroyl spermidine (C3); N1(E),N5(Z),N10(E)-tri-*p*-coumaroyl spermidine (C4); N1(E),N5(E),N10(E)-tri-*p*-coumaroyl spermidine (C5); N1(Z),N10(Z)-di-*p*-coumaroyl spermidine (C6); N1(Z),N10(E)-di-*p*-coumaroyl spermidine (C7); N1(E),N10(Z)-di-*p*-coumaroyl spermidine (C8); N1(E),N10(E)-di-*p*-coumaroyl spermidine (C9); and N1-*p*-coumaroyl-N5,N10-di-caffeoyl spermidine (C10). Compound C1 was identified in loquat honey and blueberry honey; compounds C2–C5 in apple honey and loquat honey; compounds C6–C9 in apple honey and blueberry honey; and compounds C5 and C10 in blueberry honey.

Compound C1 displayed a quasi-molecular ion at m/z 600 [M+H]^+^ (C_34_H_37_N_3_O_7_) (Table 1) and yielded characteristic MS/MS fragments at m/z 438, 292, 275, 204, and 147 (Figure 2). The quasi-molecular ion at m/z 600 generated fragment ions at m/z 438, m/z 292, and m/z 147, indicating losses of 162 amu (caffeoyl) and two of 146 amu (*p*-coumaroyl residue). The m/z 147 fragment from i-cleavage further validated the presence of *p*-coumaroyl residues. Therefore, compound C1 was identified as N1,N10-di-*p*-coumaroyl-N5-caffeoyl spermidine.

Compounds C2, C3, C4, and C5 exhibited the same quasi-molecular ion at m/z 584 [M+H]^+^ (C_34_H_37_N_3_O_6_) (Table 1) and yielded characteristic MS/MS fragments at m/z 438, 292, 275, 204, and 147 (Figure 2), indicating that they are isomers. The quasi-molecular ion at m/z 584 generated fragment ions at m/z 438, m/z 292, and m/z 147, indicating three 146 amu losses (*p*-coumaroyl residues) in succession. The m/z 147 fragment from i-cleavage further validated the presence of p-coumaroyl residues. Based on their UV λ max values at 269, 279, 290, and 298 nm, compounds C2, C3, C4, and C5 were identified, respectively, as N1(Z),N5(Z),N10(Z)-tri-*p*-coumaroyl spermidine, N1(Z),N5(Z),N10(E)-tri-*p*-coumaroyl spermidine, N1(E),N5(Z),N10(E)-tri-*p*-coumaroyl spermidine, and N1(E),N5(E),N10(E)-tri-*p*-coumaroyl spermidine. These compounds were further validated by comparing the retention times, UV λ max values, and MS/MS spectra with the standards.

Compounds C6, C7, C8, and C9 showed the same quasi-molecular ion at m/z 438 (C_25_H_31_N_3_O_4_) (Table 1) and yielded three MS/MS fragments at m/z 292, 204, and 147 (Figure 2), indicating that they are isomers. The quasi-molecular ion at m/z 438 generated fragment ions at m/z 420 and m/z 292, indicating losses of 18 amu (water) and 146 amu (*p*-coumaroyl residues). The m/z 147 fragment from i-cleavage further validated the presence of *p*-coumaroyl residues. Based on their UV λ max values at 274, 284, 292, and 306 nm, compounds C6, C7, C8, and C9 were identified, respectively, as N1(Z),N10(Z)-di-*p*-coumaroyl spermidine; N1(Z),N10(E)-di-*p*-coumaroyl spermidine; N1(E),N10(Z)-di-*p*-coumaroyl spermidine; and N1(E),N10(E)-di-*p*-coumaroyl spermidine.

Compound C10 exhibited a quasi-molecular ion at m/z 616 [M+H]^+^ (C_34_H_37_N_3_O_8_) (Table 1) and yielded characteristic MS/MS fragments at m/z 454, 308, 204, and 163 (Figure 2). The quasi-molecular ion at m/z 616 generated fragment ions at m/z 454, m/z 308, and m/z 163, indicating losses of 162 amu (caffeoyl), 146 amu (*p*-coumaroyl residues), and 162 amu (caffeoyl). The m/z 163 fragment from i-cleavage further validated the presence of caffeoyl residues. Therefore, compound C10 was identified as N1-*p*-coumaroyl-N5,N10-di-caffeoyl spermidine.

The phenolamide composition showed remarkable selectivity among the five fruit monofloral honeys. Spermidine-derived phenolamides were mainly distributed in loquat, apple, and blueberry honeys, whereas they were not detected in citrus honey. Apple honey and loquat honey were characterized by the presence of multiple tri-p-coumaroyl spermidine isomers (C2–C5), while blueberry honey contained relatively more structurally diverse phenolamides, including both di-p-coumaroyl spermidines (C6–C9) and caffeoyl-containing derivatives (C1 and C10). These differences suggest that the biosynthesis and accumulation of phenolamides may vary substantially among different fruit plants. Since phenolamides are closely associated with plant secondary metabolism and stress response pathways, their selective occurrence patterns may provide valuable phytochemical information for distinguishing fruit monofloral honeys and evaluating their botanical origins.

#### 3.1.4. Flavonoid Glycosides

Five flavonoid glycosides were identified, namely hyperoside (quercetin-3-O-galactoside) (D1), isorhamnetin-3-O-neohesperidoside (D2), kaempferol-3-O-sophoroside (D3), kaempferol-3-O-(2″-O-glucosyl)-rutinoside (D4), and isorhamnetin-3-O-vicianoside (D5). Compound D1 was identified in loquat honey; compound D2 in apple honey and blueberry honey; and compounds D3–D5 in blueberry honey.

Compound D4 exhibited a quasi-molecular ion at m/z 757 (Figure 2). The loss of a rhamnosyl moiety produced a fragment ion at m/z 611 from this precursor ion. Subsequently, the ion at m/z 611 underwent successive losses of one and then two glucosyl residues, giving rise to fragments at m/z 449 and m/z 287, indicating that the compound was a di-glucosyl-rhamnosyl derivative of kaempferol (Figure 2). Therefore, compound D4 was finally identified as kaempferol-3-O-(2’’-O-glucosyl)-rutinoside.

Compound D5 exhibited a quasi-molecular ion m/z 611 (Figure 2). The quasi-molecular ion at m/z 611 further lost a pentosyl residue (132 amu) and then a glucosyl residue (162 amu), yielding a fragment ion at m/z 479 and m/z 317. Thus, compound D5 was identified as isorhamnetin-3-O-vicianoside.

Compared with the flavonoids, flavonoid glycosides exhibited a more selective distribution pattern among the fruit monofloral honeys. Blueberry honey showed the highest diversity of flavonoid glycosides, particularly glycosylated derivatives of kaempferol and isorhamnetin (D2–D5), suggesting a relatively complex glycosylated flavonoid profile associated with *Vaccinium* floral sources. In contrast, hyperoside (D1) was only detected in loquat honey, indicating potential honey-type specificity. The differences in glycosylation patterns among honey types may reflect variations in flavonoid glycosyltransferase activity and secondary metabolic pathways in different fruit plants. These glycosylated flavonoids may therefore provide complementary phytochemical markers for distinguishing fruit monofloral honeys and evaluating their botanical origins.

#### 3.1.5. Terpenoids

Four terpenoids were identified, namely phaseic acid (E1), *trans*, *trans*-abscisic acid (E2), *cis*, *trans*-abscisic acid (E3), and callunene (E4), with their occurrence and relative abundances varying significantly among different honey types. Compound E1 was found in blueberry and pomegranate honeys; compound E2 in citrus honey; compound E3 in apple, pomegranate, and citrus honeys; and compound E4 in blueberry honey. Compounds E1–E4 have been identified in our previous study using mass spectrometry; these compounds are produced by the degradation of carotenoids and are all characterized by a 3,5,5-trimethylcyclohex-2-enic structure (Supplementary Figure S2).

The terpenoid profiles showed pronounced differences among the five fruit monofloral honeys and appeared to exhibit stronger floral specificity than several other phytochemical classes. Blueberry honey was characterized by the occurrence of both phaseic acid (E1) and callunene (E4), whereas citrus and pomegranate honeys mainly contained abscisic acid-related derivatives (E2 and E3). Since these compounds are associated with carotenoid degradation pathways, their differential distribution patterns may reflect variations in carotenoid metabolism among different fruit plants. In particular, the selective occurrence of callunene in blueberry honey suggests its potential usefulness as a characteristic marker for Vaccinium-derived honey. These results further indicate that terpenoid-related metabolites may contribute substantially to the chemical differentiation and botanical origin evaluation of fruit monofloral honeys.

#### 3.1.6. Alkaloids

An alkaloid was identified, namely caffeine (F1). Compound F1 exhibited a quasi-molecular ion at m/z 195 [M+H]^+^ (C_8_H_10_N_4_O_2_) (Figure 2). The quasi-molecular ion at m/z 195 [M+H]^+^ produced fragment ions at m/z 138, which resulted from the cleavage of the N–C bond and the loss of C_2_H_2_NO (57 Da), a well-known fragmentation pathway of caffeine involving the loss of a methylamide group. The subsequent fragment ions at m/z 123 and m/z 110 were generated through further demethylation and CO loss from the m/z 138 ion, respectively. Lower-mass fragments such as m/z 97, 83, 69, and 56 were produced by successive ring opening and N–C bond cleavage within the purine skeleton, forming stable iminium- and azacyclic-type cations. The fragmentation pattern of compound F1 is therefore consistent with the typical MS behavior of caffeine, characterized by sequential N-dealkylation, carbonyl elimination, and heterocyclic ring cleavage processes, leading to nitrogen-containing fragment ions. This component was further validated by comparing the retention time, UV λ max, and MS/MS spectra with commercial standards.

Compared with other phytochemical classes identified in this study, alkaloid-related compounds were relatively limited, with caffeine being exclusively detected in citrus honey. The selective occurrence of caffeine suggests a strong association with Citrus floral origins and highlights its potential value as a characteristic marker for citrus honey authentication. As alkaloids are relatively uncommon in honey, the presence of caffeine may reflect specific nitrogen-containing secondary metabolic pathways in Citrus plants.

### 3.2. Characteristic Profiles of Raw Honey Samples

#### 3.2.1. Loquat Honey

The chromatographic profile of loquat honey is presented in Figure 1A and Supplementary Figure S3. Thirteen characteristic phytochemicals were detected in loquat honey, including chlorogenic acid (A1), isochlorogenic acid (A2), 3-O-p-coumaroylquinic acid (A3), 5-O-feruloylquinic acid (A4), anisic acid (A5), hyperoside (D1), diosmetin (B1), 8-methoxykaempferol (B2), and several phenolamides (C1–C5). Among them, anisic acid (A5) represented the predominant constituent, with an average concentration of 7.25 mg/kg (3.95–17.13 mg/kg), accounting for nearly 35% of the total quantified phytochemicals (Figure 1A and Supplementary Table S3). The stable occurrence and relatively high abundance of anisic acid suggest its potential applicability as a characteristic marker of loquat honey.

#### 3.2.2. Pomegranate Honey

The phytochemical composition of pomegranate honey is illustrated in Figure 1B and Supplementary Figure S3. Six major compounds were identified, namely phaseic acid (E1), methyl syringate (A8), cis, trans-abscisic acid (E3), quercetin (B6), luteolin (B7), and apigenin (B8). Methyl syringate was the dominant compound in pomegranate honey, exhibiting an average level of 4.96 mg/kg (2.35–9.52 mg/kg) and contributing approximately 33.7% of the total phytochemical content (Supplementary Table S3). These results indicate that methyl syringate may serve as a representative phytochemical indicator for pomegranate honey authentication.

#### 3.2.3. Citrus Honey

Figure 1C and Supplementary Figure S3 show the characteristic HPLC profile of citrus honey. Seven phytochemicals were characterized in citrus honey samples, namely caffeine (F1), methyl syringate (A8), trans, trans-abscisic acid (E2), cis, trans-abscisic acid (E3), 5-methoxypinobanksin (B3), pinocembrin (B4), and chrysin (B5). Among these compounds, caffeine exhibited the highest abundance, with an average concentration of 10.48 mg/kg (4.40–16.72 mg/kg), corresponding to approximately 77.2% of the total quantified phytochemicals (Supplementary Table S3). The predominance of caffeine suggests that it could be considered a characteristic component of citrus honey.

#### 3.2.4. Apple Honey

The characteristic chromatographic profile of apple honey is presented in Figure 1D and Supplementary Figure S3. A total of sixteen phytochemicals were detected in apple honey samples, including 4-hydroxybenzoic acid (A6), cinnamic acid (A7), methyl syringate (A8), cis, trans-abscisic acid (E3), isorhamnetin-3-O-neohesperidoside (D2), kaempferol (B9), 8-methoxykaempferol (B2), pinocembrin (B4), and several phenolamides (C2–C9). Among these compounds, cinnamic acid and methyl syringate represented the major phytochemical constituents, together contributing approximately 60.4% of the total quantified phytochemicals (Supplementary Table S3). In particular, cinnamic acid exhibited the highest abundance, with an average concentration of 15.89 mg/kg honey, accounting for nearly 50.1% of the total phytochemical content. Methyl syringate was consistently detected in all apple honey samples, with a mean concentration of 3.27 mg/kg. The predominance and stable occurrence of cinnamic acid and methyl syringate suggest that these compounds may serve as characteristic markers for apple honey authentication.

#### 3.2.5. Blueberry Honey

The phytochemical composition of blueberry honey is shown in Figure 1E and Supplementary Figure S3. Fifteen characteristic compounds were identified, including 4-hydroxybenzoic acid (A6), phaseic acid (E1), methyl syringate (A8), callunene (E4), isorhamnetin-3-O-neohesperidoside (D2), isorhamnetin-3-O-vicianoside (D5), kaempferol-3-O-sophoroside (D3), kaempferol-3-O-(2″-O-glucosyl)-rutinoside (D4), several spermidine derivatives (C1, C5–C10), and other phenolamide-related compounds. Four major phytochemicals, namely phaseic acid (E1), methyl syringate (A8), isorhamnetin-3-O-neohesperidoside (D2), and callunene (E4), together accounted for approximately 51.7% of the total quantified phytochemicals in blueberry honey (Supplementary Table S3). Among them, phaseic acid represented the dominant constituent, with an average concentration of 1.80 mg/kg and a concentration range of 0.54–3.96 mg/kg. In addition, methyl syringate, isorhamnetin-3-O-neohesperidoside, and callunene were consistently detected in all blueberry honey samples, with average concentrations of 2.32, 6.59, and 8.27 mg/kg, respectively. The combined occurrence of these compounds highlights the distinctive phytochemical characteristics of blueberry honey and supports their potential applicability as characteristic authenticity markers.

Taken together, the five monofloral honeys exhibited clearly differentiated phytochemical composition patterns, with each honey defined by one or a limited number of dominant compounds, showing high species dependence. Loquat honey showed a strongly anisic acid-dominated profile, whereas pomegranate honey was consistently characterized by the predominance of methyl syringate. Citrus honey displayed a highly distinctive pattern marked by the overwhelming abundance of caffeine, setting it apart from all other honey types. Apple honey was defined by the combined enrichment of cinnamic acid and methyl syringate, forming a dual-marker feature unique to this honey. In contrast, blueberry honey presented a more complex but stable phytochemical signature, characterized by the concurrent occurrence of phaseic acid, methyl syringate, isorhamnetin-3-O-neohesperidoside, and callunene. These stable and honey-specific phytochemical signatures provide a robust chemical basis for differentiating botanical origins and, importantly, enable the development of reliable criteria for honey authenticity assessment. To further visualize the overall phytochemical differences among the investigated monofloral honey samples, PCA was performed based on the quantitative phytochemical data. As shown in Supplementary Figure S4, the five monofloral honey types exhibited clear clustering trends according to their characteristic phytochemical compositions, further supporting the differentiation of botanical origins based on characteristic compounds and HPLC fingerprints.

### 3.3. HPLC Fingerprints of Five Monofloral Honeys

**Figure 3:**
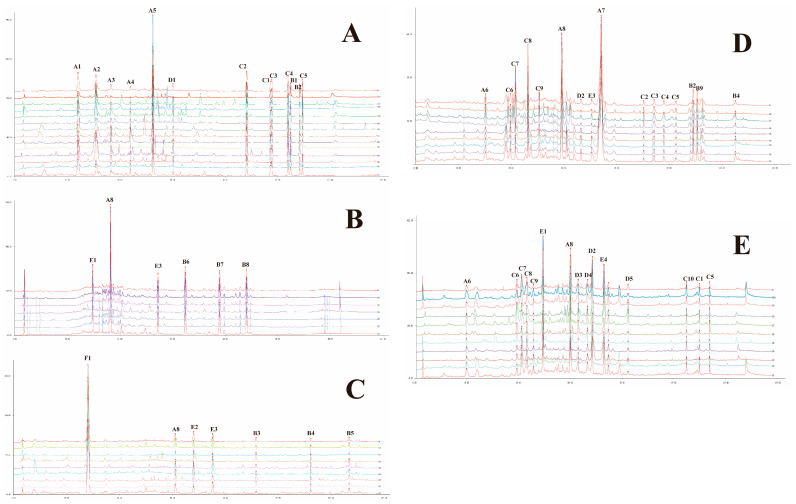
HPLC fingerprints of five monofloral raw honey samples: (**A**) loquat honey; (**B**) pomegranate honey; (**C**) citrus honey; (**D**) apple honey; (**E**) blueberry honey. Notes. A1: chlorogenic acid; A2: isochlorogenic acid; A3: 3-O-p-coumaroylquinic acid; A4: 5-O-feruloylquinic acid; A5: anisic acid; A6: 4-hydroxybenzoic acid; A7: cinnamic acid; A8: methyl syringate; B1: diosmetin; B2: 8-methoxy kaempferol; B3: 5-methoxy pinobanksin; B4: pinocembrin; B5: chrysin; B6: quercetin; B7: luteolin; B8: apigenin; B9: kaempferol; C1: N1,N10-di-*p*-coumaroyl-N5-caffeoyl spermidine; C2: N1(Z),N5(Z),N10(Z)-tri-*p*-coumaroyl spermidine; C3: N1(Z),N5(Z),N10(E)-tri-*p*-coumaroyl spermidine; C4: N1(E),N5(Z),N10(E)-tri-*p*-coumaroyl spermidine; C5: N1(E),N5(E),N10(E)-tri-*p*-coumaroyl spermidine; C6: N1(Z),N10(Z)-di-*p*-coumaroyl spermidine; C7: N1(Z),N10(E)-di-*p*-coumaroyl spermidine; C8: N1(E),N10(Z)-di-*p*-coumaroyl spermidine; C9: N1(E),N10(E)-di-*p*-coumaroyl spermidine; C10: N1-*p*-coumaroyl-N5,N10-di-caffeoyl spermidine; D1: hyperoside (quercetin-3-O-galactoside); D2: isorhamnetin-3-O-neohesperidoside; D3: kaempferol-3-O-sophoroside; D4: kaempferol-3-O-(2″-O-glucosyl)-rutinoside; D5: isorhamnetin-3-O-vicianoside; E1: phaseic acid; E2: trans, trans-abscisic acid; E3: cis, trans-abscisic acid; E4: callunene; F1: caffeine.

The HPLC fingerprint refers to a chromatographic analysis applied to honey samples after appropriate pretreatment. This method provides multi-component information and can be used to evaluate the quality characteristics of samples. Figure 3 displays the HPLC fingerprints of the five monofloral honeys, indicating that the chromatographic peak similarities across all honey samples exceeded 0.8. The fingerprint revealed unique characteristic peaks for these five monofloral honeys. These established standard fingerprints serve as non-targeted criteria for assessing the authenticity of commercial honey samples.

### 3.4. Authenticity Assessments of Commercial Honey Samples

Based on the databases of raw honeys, the evaluation criteria for genuine pure honey are as follows. Firstly, the HPLC profile of a commercial sample should be highly similar to the HPLC fingerprint profiles of genuine raw honeys. Secondly, the content of characteristic compounds should meet the proposed threshold levels. These thresholds were determined based on the 95% lower confidence bound of the 10th percentile calculated via parametric bootstrapping. For loquat honey, anisic acid was selected as the characteristic marker, with a proposed threshold of 3.0 mg/kg. For pomegranate honey, methyl syringate showed diagnostic value, with a threshold of 1.8 mg/kg. For citrus honey, caffeine was used as the major marker, with a threshold of 4.0 mg/kg. For apple honey, cinnamic acid and methyl syringate were selected as combined markers, with thresholds of 14.5 and 1.8 mg/kg, respectively. For blueberry honey, phaseic acid, methyl syringate, isorhamnetin-3-O-neohesperidoside, and callunene were proposed as characteristic markers, with tentative thresholds of 0.5, 0.6, 1.6, and 1.8 mg/kg, respectively. Samples with marker content below these thresholds may require further verification for possible adulteration or botanical mislabeling. The above content thresholds were determined based on the distribution of characteristic phytochemicals in authentic honey samples, using log-normal distribution fitting and the fifth percentile as a conservative cutoff to establish tentative lower limits for authenticity assessment.

**Figure 4:**
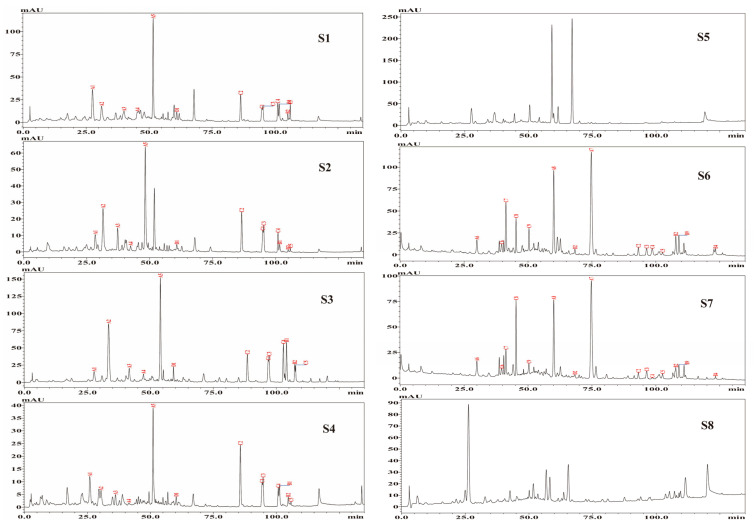

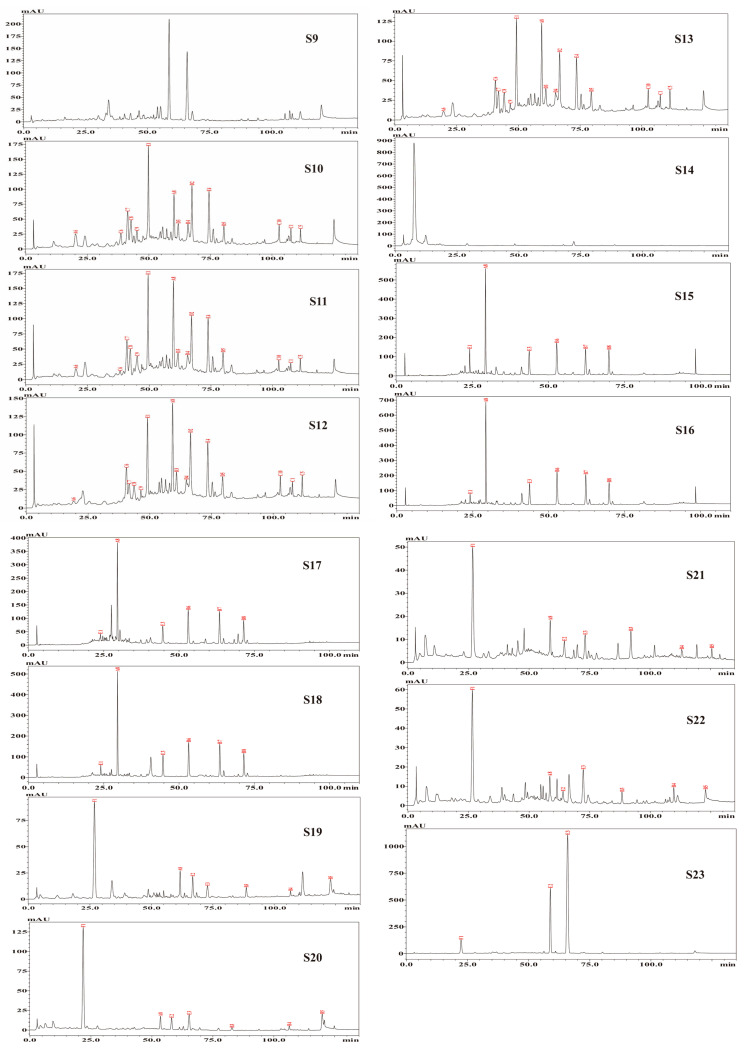
HPLC chromatograms of five types of commercial honey. Notes. S1 to S5 represent loquat commercial honey samples 1 to 5; S6 to S9 represent pomegranate honey commercial samples 1 to 4; S10 to S14 represent citrus honey commercial samples 1 to 5; S15 to S18 represent apple honey commercial samples 1 to 4; S19 to S23 represent blueberry honey commercial samples 1 to 5.

Among a total of 23 commercial samples, 18 samples exhibited chromatographic patterns that were highly similar to the corresponding patterns of raw honeys and simultaneously met the minimum content requirements for the characteristic components (Table 2). These samples were therefore classified as genuine pure honeys. In contrast, the remaining samples showed mislabeling issues regarding botanical origin and potential adulteration with sugar syrups. For example, sample S5, labeled as loquat honey, was indeed filled with honey of uncertain botanical origin (Figure 4(S5); Figure 3A). Samples S17 and S18, labeled as apple honey, had no detectable levels of cinnamic acid or methyl syringate, both of which are the characteristic markers for authentic apple honey, suggesting that these samples may not have been genuine apple honey (Figure 4(S17,S18); Figure 3D). Sample S23 had abnormal chromatographic peaks with a retention time of 8 min and did not contain any characteristic components (Figure 4(S23); Figure 3E). This commercial honey type should be added to syrups that are specially designed only for adulteration. Thus, we propose a compelling and innovative solution to differentiate botanical origins and assess honey authenticity.

## 4. Discussion

The phytochemical differences observed among the five fruit monofloral honeys appear to be closely associated with variations in floral secondary metabolism among different fruit plants. Phenolic acids, flavonoids, phenolamides, terpenoids, and alkaloid-related compounds exhibited distinct distribution patterns among the honey types, suggesting that these metabolites may partially reflect the biochemical characteristics of their botanical origins. Several compounds, including anisic acid in loquat honey, methyl syringate in pomegranate honey, caffeine in citrus honey, and phaseic acid and callunene in blueberry honey, showed relatively strong honey-type specificity and may therefore serve as potential characteristic markers for fruit monofloral honey differentiation and authenticity evaluation. Compared with conventional physicochemical parameters, these low-abundance phytochemicals may provide more direct information regarding floral source-related metabolic characteristics.

Our research elucidated the unique chemical compositions of five fruit monofloral honeys. In total, 37 phytochemical compounds were identified; notably, to the best of our knowledge, 11 compounds have not previously been reported in honey, namely 3-O-*p*-coumaroylquinic acid; 5-O-feruloylquinic acid; 5-methoxypinobanksin; N1,N10-di-*p*-coumaroyl-N5-caffeoyl spermidine; N1(Z),N10(Z)-di-*p*-coumaroyl spermidine; N1(Z),N10(E)-di-*p*-coumaroyl spermidine; N1(E),N10(Z)-di-*p*-coumaroyl spermidine; N1(E),N10(E)-di-*p*-coumaroyl spermidine; N1-*p*-coumaroyl-N5,N10-di-caffeoyl spermidine; kaempferol-3-O-(2′′-O-glucosyl)-rutinoside; and isorhamnetin-3-O-vicianoside.

As floral markers, the phytochemicals identified in this study provide a chemical basis for interpreting the biological functions of different monofloral honeys. Loquat honey, rich in anisic acid, has been linked to respiratory-soothing and anti-inflammatory properties [23]. Anisic acid is known for its antimicrobial, anti-inflammatory, and antioxidant activity [24], which may partially explain the long-standing use of loquat honey in alleviating cough and throat discomfort [25]. Pomegranate honey contained high levels of methyl syringate, a compound recognized for its strong antioxidant and antibacterial effects [26]. Methyl syringate is also a precursor of leptosperin [27], the functional marker of Manuka honey, suggesting that pomegranate honey may share similar free radical scavenging and antimicrobial potential [28]. For citrus honey, the remarkably high concentration of caffeine is particularly noteworthy. Caffeine exerts neurostimulatory effects, enhancing alertness and cognitive function; consequently, caffeine-infused citrus honey presents a viable alternative to sweetened coffee. Caffeine has also been widely reported to exert antimicrobial, anti-biofilm, and antifungal activity [29]. Therefore, its elevated presence in citrus honey may reinforce the honey’s ability to inhibit microbial growth and suppress pathogenic biofilm formation. Apple honey was distinguished by abundant cinnamic acid and methyl syringate; cinnamic acid is a phenylpropanoid with broad-spectrum antimicrobial and anti-inflammatory properties [30], supporting the characteristic bioactivity of this honey. Blueberry honey displayed a distinctive combination of phaseic acid, methyl syringate, isorhamnetin-3-O-neohesperidoside, and callunene, collectively suggesting enhanced antioxidant and anti-inflammatory potential compared with the other monofloral honeys [31]. Ultimately, the highly species-dependent phytochemicals seem to impart unique functional properties to each honey type.

Previous studies on monofloral honey authentication have mainly focused on melissopalynology, physicochemical parameters, volatile compounds, stable isotope analysis, or individual phytochemical markers. Compared with these approaches, the present study combined characteristic phytochemicals with chromatographic fingerprint analysis to establish a preliminary authenticity evaluation strategy for multiple fruit monofloral honeys simultaneously. In addition, several characteristic compounds exhibiting relatively strong honey-type specificity were identified, further supporting the potential application of phytochemical profiling in botanical origin differentiation and honey authenticity evaluation.

Beyond compositional characterization, the present study demonstrates that combining the quantitative determination of characteristic phytochemicals with HPLC fingerprint analysis provides a robust and practical strategy for monofloral honey authentication. Unlike approaches relying on a single marker or general physicochemical parameters, the integration of multi-marker quantification and non-targeted fingerprint similarity enables both specificity and tolerance toward natural variation among authentic samples. In this study, each monofloral honey was defined by one or a limited number of dominant phytochemicals with stable content ranges across geographically distinct raw samples, allowing the establishment of minimum threshold values for authenticity assessment. When applied to commercial honeys, this dual-criteria approach effectively differentiated genuine products from adulterated or mislabeled samples, including cases involving blended honeys or specially designed syrup adulterants. These findings indicate that phytochemical-based authenticity evaluation, supported by quantitative thresholds and chromatographic fingerprints, represents a feasible and transferable framework for quality control in monofloral honeys, particularly for honey types lacking established international standards. From a practical perspective, the long HPLC gradient (135 min) ensured the adequate separation of diverse phytochemicals but may limit its application in routine high-throughput analysis.

The five fruit crops investigated in this study are closely associated with insect pollination, and honeybees play an important role in supporting fruit set, yields, and quality. During this process, honey produced from these fruit nectar sources represents an important by-product and provides an additional income source for beekeepers. Therefore, the significance of this study extends beyond compositional characterization and authenticity evaluation. By clarifying the characteristic phytochemicals of fruit monofloral honeys and establishing corresponding quality control criteria, this work may help to improve market regulation and protect the value of authentic monofloral honey products. A reliable method for quality differentiation could support higher market value for genuine monofloral honeys, thereby improving the economic return to beekeepers and encouraging apicultural activities in fruit-growing regions. For major fruit crops such as citrus, this is particularly important because apiculture contributes not only to monofloral honey production but also to pollination services that support agricultural sustainability. Thus, phytochemical-based quality evaluation of monofloral honeys may link honey quality control with beekeeper incentives, crop pollination services, and the sustainable development of fruit-based agroecosystems.

## 5. Conclusions

In this study, regarding the five fruit monofloral honeys, we identified 37 phytochemical compounds, among which 11 compounds appear to be reported in honey for the first time. Quantitative analysis revealed distinct characteristic markers for each honey type. Anisic acid was identified as the characteristic marker for loquat honey, with average content of 7.25 mg/kg; methyl syringate (average content, 4.96 mg/kg) for pomegranate honey; caffeine for citrus honey (average content, 10.48 mg/kg); cinnamic acid and methyl syringate for apple honey, with average content of 15.89 and 3.27 mg/kg, respectively; and phaseic acid, methyl syringate, isorhamnetin-3-O-neohesperidoside, and callunene for blueberry honey, with average content of 1.80, 2.32, 6.59, and 8.27 mg/kg, respectively. These characteristic phytochemicals could serve as potential marker compounds for botanical origin discrimination and contribute to the proposed authenticity assessment strategy for fruit monofloral honeys.

This study proposes a novel solution to assess the authenticity of these monofloral honeys by combining target characteristic compounds with non-target fingerprints. The identification of these compounds may provide preliminary support for the authenticity identification of honey and suggest the potential of honey as a functional food. Future studies should focus on the bioactivity of these characteristic components to advance the scientific research and applications of honey.

## Figures and Tables

**Table 1:** Compounds identified in five types of honey and their UV values and characteristics.

Peak	Mw	UV λ Max (nm)	Formula	m/z of Main Fragments, MS2	Compound Identified	Honey
A1	354	326	C_16_H_18_O_9_	191	Chlorogenic acid ^#^	Loquat
A2	354	324	C_16_H_18_O_9_	191	Isochlorogenic acid	Loquat
A3 *	338	236,311	C_16_H_18_O_8_	191	3-O-*p*-Coumaroylquinic acid ^#^	Loquat
A4 *	368	233,324	C_17_H_20_O_9_	191	5-O-Feruloylquinic acid ^#^	Loquat
A5	152	256	C_8_H_8_O_3_	51, 77, 94, 109, 114, 135	Anisic acid (4-Methoxybenzoic acid) ^#^	Loquat
A6	138	255	C_7_H_6_O_3_	75, 93	4-Hydroxybenzoic acid ^#^	Apple, Blueberry
A7	148	275	C_9_H_8_O_2_	77, 103	Cinnamic acid ^#^	Apple
A8	212	275	C_10_H_12_O_5_	77, 93, 111, 120, 139, 148, 154, 166, 181	Methyl syringate ^#^	Apple, Blueberry, Citrus, Pomegranate
B1	300	267,348	C_16_H_12_O_6_	133, 153, 203, 229, 258, 286	Diosmetin ^#^	Loquat
B2	316	271,374	C_16_H_12_O_7_	65, 92, 121, 153, 179, 203, 229, 274	8-Methoxykaempferol ^#^	Apple, Loquat
B3 *	286	232,287	C_16_H_14_O_5_	91, 167, 213, 226, 241, 269	5-Methoxypinobanksin ^#^	Citrus
B4	256	290	C_15_H_12_O_4_	103, 131, 153, 173, 207, 226	Pinocembrin ^#^	Citrus
B5	254	268,313	C_15_H_10_O_4_	68, 77, 103, 129, 153, 165, 181, 209	Chrysin ^#^	Apple, Citrus
B6	302	254,295,368	C_15_H_10_O_7_	68, 81, 109, 137, 153, 183, 201, 229, 257	Quercetin ^#^	Pomegranate
B7	286	349	C_15_H_10_O_6_	68, 89, 135, 153, 185, 213, 241, 269	Luteolin ^#^	Pomegranate
B8	270	267,338	C_15_H_10_O_5_	68, 91, 119, 153, 197, 243	Apigenin ^#^	Pomegranate
B9	286	266,296,366	C_15_H_10_O_6_	95, 121, 137, 153, 165, 213, 231, 258	Kaempferol ^#^	Apple
C1 *	599	292	C_34_H_37_N_3_O_7_	147, 204, 218, 292, 420, 438	N1,N10-di-*p*-coumaroyl-N5-caffeoyl spermidine	Blueberry, Loquat
C2	583	269	C_34_H_37_N_3_O_6_	147, 204, 218, 292, 420, 438	N1(Z),N5(Z),N10(Z)-tri-*p*-coumaroyl spermidine	Apple, Loquat
C3	583	279	C_34_H_37_N_3_O_6_	147, 204, 218, 292, 420, 438	N1(Z),N5(Z),N10(E)-tri-*p*-coumaroyl spermidine	Apple, Loquat
C4	583	290	C_34_H_37_N_3_O_6_	147, 204, 218, 292, 420, 438	N1(E),N5(Z),N10(E)-tri-*p*-coumaroyl spermidine	Apple, Loquat
C5	583	298	C_34_H_37_N_3_O_6_	147, 204, 218, 292, 420, 438	N1(E),N5(E),N10(E)-tri-*p*-coumaroyl spermidine	Apple, Blueberry, Loquat
C6 *	437	274	C_25_H_31_N_3_O_4_	147, 204, 275, 292	N1(Z),N10(Z)-di-*p*-coumaroyl spermidine	Apple, Blueberry
C7 *	437	284	C_25_H_31_N_3_O_4_	147, 204, 275, 292	N1(Z),N10(E)-di-*p*-coumaroyl spermidine	Apple, Blueberry
C8 *	437	292	C_25_H_31_N_3_O_4_	147, 204, 275, 292	N1(E),N10(Z)-di-*p*-coumaroyl spermidine	Apple, Blueberry
C9 *	437	306	C_25_H_31_N_3_O_4_	147, 204, 275, 292	N1(E),N10(E)-di-*p*-coumaroyl spermidine	Apple, Blueberry
C10 *	615	296	C_34_H_37_N_3_O_8_	147, 163, 204, 308, 454	N1-*p*-coumaroyl-N5,N10-di-caffeoyl spermidine	Blueberry
D1	464	354	C_21_H_20_O_12_	303, 465	Hyperoside (quercetin-3-O-galactoside) ^#^	Loquat
D2	624	355	C_28_H_32_O_16_	317, 479	Isorhamnetin-3-O-neohesperidoside ^#^	Apple, Blueberry
D3	610	348	C_27_H_30_O_16_	287, 449	Kaempferol-3-O-sophoroside ^#^	Blueberry
D4 *	756	348	C_33_H_40_O_20_	287, 449, 611	Kaempferol-3-O-(2’’-O-glucosyl)- rutinoside	Blueberry
D5 *	610	355	C_27_H_30_O_16_	317, 479	Isorhamnetin-3-O-vicianoside	Blueberry
E1	280	264	C_15_H_20_O_5_	57, 83, 109, 124, 139, 168, 187, 205	Phaseic acid	Blueberry, Pomegranate
E2	264	263	C_15_H_20_O_4_	93, 107, 135, 163, 187, 209, 229, 248	*trans*, *trans*-abscisic acid ^#^	Citrus
E3	264	263	C_15_H_20_O_4_	121, 135, 159, 173, 187, 201, 229	*cis*, *trans*-abscisic acid ^#^	Apple, Pomegranate, Citrus
E4	204	282	C_13_H_16_O_2_	69, 77, 105, 121, 129, 147, 163, 187	Callunene	Blueberry
F1	194	272	C_8_H_10_N_4_O_2_	56, 69, 83, 87, 110, 123, 138	Caffeine ^#^	Citrus

Notes. *: first identified from honey; #: compound identified by comparison with commercial standards.

**Table 2:** Analysis of compound content in five types of commercial honey samples.

Loquat	A1				A2				A3			A4					A5			D1					C2				C1			C3			C4				B1			B2				C5		
S1	0.42 ± 0.04				0.25 ± 0.03				1.27 ± 0.10			1.01 ± 0.08					9.86 ± 0.74			0.74 ± 0.06					1.40 ± 0.09				1.18 ± 0.08			0.81 ± 0.07			0.85 ± 0.08				1.43 ± 0.11			0.13 ± 0.02				0.64 ± 0.05		
S2	0.15 ± 0.02				0.34 ± 0.03				0.66 ± 0.05			0.26 ± 0.03					5.61 ± 0.42			0.37 ± 0.04					1.18 ± 0.08				Nd			0.75 ± 0.06			0.58 ± 0.05				Nd			Nd				0.23 ± 0.03		
S3	0.21 ± 0.03				1.08 ± 0.09				1.24 ± 0.10			1.11 ± 0.08					11.84 ± 0.95			1.17 ± 0.09					1.98 ± 0.11				1.10 ± 0.08			1.68 ± 0.12			2.11 ± 0.15				0.46 ± 0.04			0.03 ± 0.01				0.87 ± 0.07		
S4	0.17 ± 0.02				0.11 ± 0.02				0.24 ± 0.03			0.10 ± 0.01					4.25 ± 0.30			0.26 ± 0.03					1.19 ± 0.08				2.21 ± 0.16			0.60 ± 0.05			0.43 ± 0.04				4.06 ± 0.32			0.35 ± 0.03				0.23 ± 0.03		
S5	Nd				Nd				Nd			Nd					Nd			Nd					Nd				Nd			Nd			Nd				Nd			Nd				Nd		
**Pomegranate**						**E1**							**A8**								**E3**							**B6**								**B7**							**B8**					
S6						1.90 ± 0.14							12.08 ± 0.85								2.77 ± 0.17							5.80 ± 0.35								7.61 ± 0.42							2.96 ± 0.21					
S7						1.07 ± 0.08							14.68 ± 0.92								3.16 ± 0.19							7.11 ± 0.42								10.92 ± 0.55							3.37 ± 0.23					
S8						0.61 ± 0.05							8.31 ± 0.50								1.59 ± 0.09							3.97 ± 0.22								6.63 ± 0.33							1.99 ± 0.12					
S9						1.23 ± 0.09							12.71 ± 0.81								2.14 ± 0.12							4.98 ± 0.27								7.45 ± 0.40							2.68 ± 0.17					
**Citrus**				**E1**							**A8**							**E2**						**E3**							**B3**						**B4**							**B5**				
S10				8.72 ± 0.61							1.00 ± 0.06							0.52 ± 0.03						0.33 ± 0.03							0.71 ± 0.05						0.17 ± 0.02							1.08 ± 0.08				
S11				10.29 ± 0.77							0.55 ± 0.03							0.34 ± 0.02						0.65 ± 0.04							0.48 ± 0.03						0.20 ± 0.02							1.18 ± 0.09				
S12				5.29 ± 0.37							0.60 ± 0.04							0.12 ± 0.01						0.29 ± 0.02							0.92 ± 0.06						0.19 ± 0.02							0.39 ± 0.03				
S13				5.86 ± 0.41							0.49 ± 0.03							0.07 ± 0.01						0.58 ± 0.03							0.55 ± 0.04						0.27 ± 0.03							0.56 ± 0.04				
S14				10.04 ± 0.75							Nd							0.81 ± 0.05						0.83 ± 0.05							Nd						Nd							Nd				
**Apple**		**A6**	**C6**				**C7**			**C8**				**C9**		**A8**			**D2**				**E3**			**A7**				**C2**		**C3**		**C4**				**C5**			**B2**				**B9**			**B4**
S15		0.60 ± 0.05	0.51 ± 0.04				1.77 ± 0.11			0.87 ± 0.05				0.86 ± 0.06		4.85 ± 0.34			0.22 ± 0.03				Nd			16.26 ± 1.02				0.31 ± 0.03		0.33 ± 0.03		0.44 ± 0.04				0.17 ± 0.02			0.75 ± 0.05				0.74 ± 0.05			0.24 ± 0.02
S16		0.58 ± 0.04	0.39 ± 0.03				0.82 ± 0.05			1.44 ± 0.08				0.53 ± 0.03		4.30 ± 0.30			0.15 ± 0.02				Nd			16.33 ± 1.03				0.23 ± 0.02		0.35 ± 0.03		0.10 ± 0.01				0.23 ± 0.02			0.38 ± 0.03				0.47 ± 0.03			0.13 ± 0.01
S17		Nd	Nd				Nd			Nd				Nd		Nd			Nd				Nd			Nd				Nd		Nd		Nd				Nd			Nd				Nd			Nd
S18		Nd	Nd				Nd			Nd				Nd		Nd			Nd				Nd			Nd				Nd		Nd		Nd				Nd			Nd				Nd			Nd
**Blueberry**		**A6**		**C6**				**C7**			**C8**				**C9**			**E1**				**A8**		**D3**			**D4**				**D2**		**E4**				**D5**			**C10**				**C1**			**C5**	
S19		4.18 ± 0.33		3.00 ± 0.23				6.85 ± 0.47			5.90 ± 0.35				3.02 ± 0.21			5.43 ± 0.38				4.32 ± 0.32		1.04 ± 0.07			1.27 ± 0.08				11.77 ± 0.85		16.98 ± 1.02				3.87 ± 0.28			2.18 ± 0.15				1.44 ± 0.10			0.79 ± 0.06	
S20		2.49 ± 0.19		1.69 ± 0.12				7.21 ± 0.50			5.72 ± 0.36				5.93 ± 0.39			5.51 ± 0.36				8.03 ± 0.56		1.42 ± 0.10			1.55 ± 0.09				13.36 ± 0.94		19.80 ± 1.19				4.48 ± 0.30			1.26 ± 0.09				0.93 ± 0.06			0.79 ± 0.05	
S21		1.27 ± 0.10		5.88 ± 0.41				3.55 ± 0.25			3.53 ± 0.22				1.46 ± 0.10			4.08 ± 0.29				6.80 ± 0.42		0.98 ± 0.07			0.95 ± 0.06				11.97 ± 0.84		14.47 ± 1.01				4.04 ± 0.26			1.62 ± 0.11				1.24 ± 0.08			1.03 ± 0.06	
S22		1.55 ± 0.12		5.18 ± 0.36				3.58 ± 0.25			2.98 ± 0.19				0.88 ± 0.06			3.36 ± 0.23				4.70 ± 0.31		0.55 ± 0.04			0.48 ± 0.03				8.64 ± 0.63		12.66 ± 0.84				3.60 ± 0.23			1.70 ± 0.12				1.14 ± 0.07			0.90 ± 0.05	
S23		Nd		Nd				Nd			Nd				Nd			Nd				Nd		Nd			Nd				Nd		Nd				Nd			Nd				Nd			Nd	

Note. Nd: not detected.

## Data Availability

The data used to support the findings of this study can be made available by the corresponding authors upon request.

## Supplementary Materials

The following supporting information can be downloaded at https://www.mdpi.com/article/10.3390/foods15111954/s1, Supplementary Table S1. Characterization of the analyzed raw honey samples; Supplementary Table S2. Manufacturing dates and addresses of five types of commercial honey; Supplement Table S3. Analysis of compound content in five types of raw honey samples; Supplementary Table S4. Method validation parameters for the compounds identified in five types of honey, including linearity, R^2^, LOD, LOQ, recovery, and precision (RSD%); Supplementary Figure S1. Pollen grain photomicrographs of five types of honey; Supplementary Figure S2. MS/MS spectra (ESI) and fragment cleavage patterns of phytochemicals; Supplementary Figure S3. HPLC profiles of five types of raw honey. Supplementary Figure S4. PCA score plot showing the clustering of the five monofloral honey samples according to their phytochemical profiles.
