# Supplementary material for: Authenticity Assessment of Five Monofloral Honeys Based on Phytochemical Profiles
Source: Foods. 2026 Jun 1;15(11):1954. doi: 10.3390/foods15111954 (PMC13257018; doi:10.3390/foods15111954)
Supplement: Supplementary file 1 [file foods-15-01954-s001.zip › foods-4318478-supplementary.pdf]

Supplement Table S1. Characterization of the analyzed raw honey samples

Sample	Type of honey	Botanical origin	Predominant pollen (%)	Geographical origin	Production year
R1	Loquat honey	*Eriobotrya japonica* (Thunb.) Lindl.	71	Putian, Fujian	2024
R2	Loquat honey	*Eriobotrya japonica* (Thunb.) Lindl.	73	Putian, Fujian	2024
R3	Loquat honey	*Eriobotrya japonica* (Thunb.) Lindl.	75	Putian, Fujian	2024
R4	Loquat honey	*Eriobotrya japonica* (Thunb.) Lindl.	78	Tangqi, Zhejiang	2024
R5	Loquat honey	*Eriobotrya japonica* (Thunb.) Lindl.	79	Tangqi, Zhejiang	2024
R6	Loquat honey	*Eriobotrya japonica* (Thunb.) Lindl.	76	Tangqi, Zhejiang	2024
R7	Loquat honey	*Eriobotrya japonica* (Thunb.) Lindl.	81	Wuzhong, Jiangsu	2024
R8	Loquat honey	*Eriobotrya japonica* (Thunb.) Lindl.	73	Wuzhong, Jiangsu	2024
R9	Loquat honey	*Eriobotrya japonica* (Thunb.) Lindl.	77	Wuzhong, Jiangsu	2024
R10	Loquat honey	*Eriobotrya japonica* (Thunb.) Lindl.	71	Wuzhong, Jiangsu	2024
R11	Loquat honey	*Eriobotrya japonica* (Thunb.) Lindl.	68	Wuzhong, Jiangsu	2024
R12	Loquat honey	*Eriobotrya japonica* (Thunb.) Lindl.	76	Wuzhong, Jiangsu	2024
R13	Loquat honey	*Eriobotrya japonica* (Thunb.) Lindl.	74	Wuzhong, Jiangsu	2024
R14	Apple honey	*Malus pumila Mill.*	72	Yantai, Shandong	2024
R15	Apple honey	*Malus pumila Mill.*	72	Yantai, Shandong	2024
R16	Apple honey	*Malus pumila Mill.*	74	Yantai, Shandong	2024
R17	Apple honey	*Malus pumila Mill.*	72	Luochuan, Shaanxi	2024
R18	Apple honey	*Malus pumila Mill.*	71	Luochuan, Shaanxi	2024
R19	Apple honey	*Malus pumila Mill.*	67	Yuncheng, Shanxi	2024
R20	Blueberry honey	*Vaccinium* spp.	65	Yichun, Heilongjiang	2024
R21	Blueberry honey	*Vaccinium* spp.	74	Yichun, Heilongjiang	2024
R22	Blueberry honey	*Vaccinium* spp.	68	Yichun, Heilongjiang	2024
R23	Blueberry honey	*Vaccinium* spp.	63	Weining, Guizhou	2024
R24	Blueberry honey	*Vaccinium* spp.	69	Weining, Guizhou	2024
R25	Blueberry honey	*Vaccinium* spp.	73	Bijie, Guizhou	2024
R26	Blueberry honey	*Vaccinium* spp.	71	Bijie, Guizhou	2024
R27	Blueberry honey	*Vaccinium* spp.	79	Dandong, Liaoning	2024

Sample	Type of honey	Botanical origin	Predominant pollen (%)	Geographical origin	Production year
R28	Blueberry honey	*Vaccinium* spp.	72	Dandong, Liaoning	2024
R29	Blueberry honey	*Vaccinium* spp.	78	Dandong, Liaoning	2024
R30	Pomegranate honey	*Punica granatum* L.	79	Luoyang, Henan	2024
R31	Pomegranate honey	*Punica granatum* L.	79	Luoyang, Henan	2024
R32	Pomegranate honey	*Punica granatum* L.	73	Huili, Yunnan	2024
R33	Pomegranate honey	*Punica granatum* L.	75	Huili, Yunnan	2024
R34	Pomegranate honey	*Punica granatum* L.	72	Kashgar, Xinjiang	2024
R35	Pomegranate honey	*Punica granatum* L.	78	Kashgar, Xinjiang	2024
R36	Citrus honey	*Citrus reticulata* Blanco	77	Changde, Hunan	2024
R37	Citrus honey	*Citrus reticulata* Blanco	75	Yueyang, Hunan	2024
R38	Citrus honey	*Citrus reticulata* Blanco	72	Yueyang, Hunan	2024
R39	Citrus honey	*Citrus reticulata* Blanco	77	Yichang, Hubei	2024
R40	Citrus honey	*Citrus reticulata* Blanco	79	Yichang, Hubei	2024
R41	Citrus honey	*Citrus reticulata* Blanco	79	Yibin, Sichuan	2024
R42	Citrus honey	*Citrus reticulata* Blanco	72	Yibin, Sichuan	2024
R43	Citrus honey	*Citrus reticulata* Blanco	78	Luzhou, Sichuan	2024

Supplement Table S2. Manufacturing date and address of five types of commercial honey

Brands	Date	Address
S1	2025-03-15	Changde, Hunan,
S2	2025-03-20	Yichang, Hubei
S3	2025-03-28	Ganzhou, Jiangxi
S4	2025-04-05	Liuzhou, Guangxi,
S5	2025-04-12	Yibin, Sichuan
S6	2025-04-20	Luochuan, Shaanxi
S7	2025-04-28	Yuncheng, Shanxi
S8	2025-05-05	Lingbao, Henan
S9	2025-05-12	Qingyang, Gansu
S10	2025-05-18	Dalian, Liaoning
S11	2025-05-05	Dandong, Liaoning
S12	2025-05-12	Baishan, Jilin
S13	2025-05-18	Yichun, Heilongjiang
S14	2025-05-22	Weining, Guizhou
S15	2025-05-28	Luoyang, Henan
S16	2025-05-25	Kashgar, Xinjiang
S17	2025-06-01	Huaiyuan, Anhui
S18	2025-06-08	Huili, Sichuan
S19	2025-06-12	Huayin, Shaanxi
S20	2025-06-18	Putian, Fujian
S21	2025-02-25	Fuzhou, Fujian
S22	2025-03-05	Tangqi, Zhejiang
S23	2025-03-10	Dongshan, Jiangsu

Supplement Table S3. Analysis of compound contents in five type raw honey samples (mg/kg)

Loquat	A1	A2	A3	A4	A5	D1	C2	C1	C3	C4	B1	B2	C5
R1	1.00 ± 0.06	0.33 ± 0.04	0.68 ± 0.08	0.92 ± 0.08	4.41 ± 0.33	0.40 ± 0.03	2.00 ± 0.18	1.92 ± 0.22	1.23 ± 0.15	1.38 ± 0.20	1.74 ± 0.15	0.24 ± 0.03	0.75 ± 0.07
R2	0.27 ± 0.02	0.19 ± 0.03	0.76 ± 0.10	0.66 ± 0.04	5.02 ± 0.69	1.26 ± 0.16	1.29 ± 0.10	1.11 ± 0.13	0.68 ± 0.06	0.73 ± 0.05	2.97 ± 0.44	0.15 ± 0.01	0.76 ± 0.04
R3	0.68 ± 0.06	0.80 ± 0.08	1.08 ± 0.15	0.28 ± 0.02	6.74 ± 0.98	0.95 ± 0.13	2.75 ± 0.34	2.33 ± 0.33	1.55 ± 0.10	2.58 ± 0.31	Nd	0.17 ± 0.01	0.60 ± 0.08
R4	0.47 ± 0.05	0.62 ± 0.05	1.14 ± 0.16	0.45 ± 0.05	12.66 ± 1.40	1.98 ± 0.24	2.89 ± 0.37	2.88 ± 0.15	2.11 ± 0.16	2.93 ± 0.18	3.54 ± 0.29	Nd	2.10 ± 0.20
R5	0.69 ± 0.05	0.26 ± 0.01	1.43 ± 0.20	0.31 ± 0.03	6.61 ± 0.50	0.90 ± 0.12	2.50 ± 0.15	2.67 ± 0.39	1.66 ± 0.10	3.11 ± 0.44	3.96 ± 0.48	0.51 ± 0.05	3.87 ± 0.37
R6	0.38 ± 0.04	0.39 ± 0.04	1.42 ± 0.18	3.06 ± 0.36	3.95 ± 0.34	1.01 ± 0.12	1.67 ± 0.12	1.37 ± 0.08	0.95 ± 0.05	0.83 ± 0.05	1.04 ± 0.10	0.10 ± 0.01	0.33 ± 0.05
R7	1.23 ± 0.18	0.24 ± 0.02	1.31 ± 0.16	1.10 ± 0.08	4.15 ± 0.41	0.85 ± 0.10	2.13 ± 0.13	1.77 ± 0.11	1.28 ± 0.08	1.01 ± 0.07	1.02 ± 0.08	0.11 ± 0.01	0.32 ± 0.04
R8	0.83 ± 0.07	0.40 ± 0.04	0.84 ± 0.06	0.86 ± 0.06	4.68 ± 0.37	0.51 ± 0.03	1.88 ± 0.23	1.36 ± 0.08	1.16 ± 0.07	1.57 ± 0.15	Nd	0.23 ± 0.03	0.37 ± 0.04
R9	0.35 ± 0.03	0.94 ± 0.08	0.72 ± 0.05	0.76 ± 0.05	5.04 ± 0.33	2.20 ± 0.33	3.08 ± 0.29	2.30 ± 0.21	1.49 ± 0.13	1.77 ± 0.14	3.05 ± 0.27	0.12 ± 0.01	1.99 ± 0.25
R10	0.61 ± 0.05	0.86 ± 0.07	1.38 ± 0.12	0.90 ± 0.05	9.31 ± 0.81	1.08 ± 0.08	1.83 ± 0.16	1.17 ± 0.08	0.78 ± 0.05	0.62 ± 0.04	0.53 ± 0.03	0.06 ± 0.01	0.37 ± 0.04
R11	0.24 ± 0.03	0.68 ± 0.05	1.11 ± 0.08	0.31 ± 0.02	17.13 ± 1.72	2.05 ± 0.27	2.66 ± 0.22	1.48 ± 0.09	0.93 ± 0.06	2.36 ± 0.26	0.20 ± 0.02	0.25 ± 0.02	0.76 ± 0.05
R12	0.08 ± 0.01	0.08 ± 0.01	1.81 ± 0.18	1.48 ± 0.13	9.64 ± 0.77	0.78 ± 0.07	1.22 ± 0.07	1.15 ± 0.07	0.78 ± 0.05	0.79 ± 0.06	0.46 ± 0.04	0.05 ± 0.01	0.46 ± 0.04
R13	0.15 ± 0.02	0.39 ± 0.03	0.28 ± 0.02	0.14 ± 0.01	4.97 ± 0.37	0.19 ± 0.01	1.71 ± 0.14	1.46 ± 0.10	0.96 ± 0.05	0.59 ± 0.04	1.75 ± 0.12	Nd	0.89 ± 0.06

Pomegranate	E1	A8	E3	B6	B7	B8
R14	2.11 ± 0.15	7.03 ± 0.64	1.59 ± 0.09	3.15 ± 0.40	4.93 ± 0.46	1.91 ± 0.23
R15	1.76 ± 0.24	2.73 ± 0.22	3.84 ± 0.52	1.72 ± 0.11	4.18 ± 0.34	1.84 ± 0.22
R16	1.76 ± 0.11	5.24 ± 0.59	0.98 ± 0.09	2.46 ± 0.24	4.02 ± 0.52	1.57 ± 0.19
R17	2.74 ± 0.39	9.52 ± 1.00	2.35 ± 0.29	4.89 ± 0.58	6.40 ± 0.68	2.59 ± 0.29
R18	1.21 ± 0.14	2.35 ± 0.26	1.72 ± 0.10	1.71 ± 0.20	10.80 ± 1.52	2.40 ± 0.15
R19	1.49 ± 0.22	2.87 ± 0.27	1.94 ± 0.11	1.49 ± 0.18	13.37 ± 1.64	2.83 ± 0.29

Citrus	E1	A8	E2	E3	B3	B4	B5
R20	11.88 ± 0.77	0.98 ± 0.08	1.17 ± 0.12	0.31 ± 0.03	0.94 ± 0.06	0.19 ± 0.02	0.80 ± 0.06
R21	12.43 ± 0.92	0.75 ± 0.06	0.40 ± 0.03	0.83 ± 0.04	0.52 ± 0.03	0.23 ± 0.03	0.54 ± 0.05
R22	12.12 ± 0.81	1.09 ± 0.08	1.23 ± 0.15	0.33 ± 0.03	0.89 ± 0.05	0.17 ± 0.02	0.56 ± 0.05
R23	11.52 ± 0.86	0.78 ± 0.06	0.60 ± 0.07	1.26 ± 0.12	0.61 ± 0.05	0.34 ± 0.05	0.55 ± 0.05
R24	9.00 ± 0.63	0.39 ± 0.03	0.04 ± 0.01	0.59 ± 0.05	0.69 ± 0.05	0.34 ± 0.05	0.73 ± 0.05
R25	16.72 ± 1.68	Nd	0.57 ± 0.05	0.43 ± 0.04	1.21 ± 0.09	0.49 ± 0.04	0.56 ± 0.05

(continued)

Citrus	E1	A8	E2	E3	B3	B4	B5
R26	5.77 ± 0.46	0.11 ± 0.01	0.13 ± 0.01	0.18 ± 0.02	0.36 ± 0.03	0.11 ± 0.01	0.37 ± 0.03
R27	4.40 ± 0.31	0.12 ± 0.01	0.12 ± 0.01	0.19 ± 0.02	0.37 ± 0.03	0.21 ± 0.03	0.38 ± 0.03

Apple	A6	C6	C7	C8	C9	A8	D2	E3	A7	C2	C3	C4	C5	B2	B9	B4
R28	1.74 ± 0.12	1.50 ± 0.11	1.15 ± 0.10	1.48 ± 0.12	1.71 ± 0.13	3.85 ± 0.34	2.26 ± 0.25	0.22 ± 0.02	16.25 ± 1.20	0.34 ± 0.03	0.51 ± 0.04	0.43 ± 0.03	0.44 ± 0.03	0.40 ± 0.03	0.33 ± 0.02	0.39 ± 0.03
R29	1.66 ± 0.11	1.30 ± 0.09	1.29 ± 0.10	2.04 ± 0.14	1.53 ± 0.12	3.89 ± 0.31	1.79 ± 0.22	0.13 ± 0.02	16.51 ± 1.25	0.40 ± 0.03	0.47 ± 0.04	0.40 ± 0.03	0.47 ± 0.04	0.31 ± 0.02	0.20 ± 0.02	0.19 ± 0.01
R30	1.69 ± 0.11	1.02 ± 0.08	1.06 ± 0.08	1.04 ± 0.08	2.12 ± 0.16	2.75 ± 0.28	2.61 ± 0.26	0.23 ± 0.02	15.35 ± 1.12	0.28 ± 0.02	0.47 ± 0.04	0.47 ± 0.04	0.37 ± 0.03	0.32 ± 0.03	0.35 ± 0.03	0.52 ± 0.04
R31	1.50 ± 0.10	1.19 ± 0.09	1.29 ± 0.10	2.37 ± 0.15	1.42 ± 0.11	3.18 ± 0.29	1.77 ± 0.20	0.09 ± 0.01	15.94 ± 1.24	0.41 ± 0.03	0.42 ± 0.03	0.44 ± 0.03	0.46 ± 0.03	0.25 ± 0.02	0.21 ± 0.02	0.08 ± 0.01
R32	1.28 ± 0.09	1.12 ± 0.08	1.18 ± 0.09	2.42 ± 0.15	1.19 ± 0.09	1.96 ± 0.14	1.81 ± 0.15	0.01 ± 0.01	14.94 ± 1.12	0.31 ± 0.02	0.36 ± 0.03	0.44 ± 0.03	0.43 ± 0.03	0.26 ± 0.02	0.22 ± 0.02	0.06 ± 0.01
R33	1.89 ± 0.12	1.30 ± 0.10	2.60 ± 0.26	3.02 ± 0.30	1.82 ± 0.13	4.00 ± 0.32	1.70 ± 0.25	Nd	16.34 ± 1.28	0.48 ± 0.03	0.54 ± 0.04	0.33 ± 0.03	0.38 ± 0.03	0.35 ± 0.03	0.24 ± 0.02	0.17 ± 0.01

Blueberry	A6	C6	C7	C8	C9	E1	A8	D3	D4	D2	E4	D5	C10	C1	C5
R34	1.49 ± 0.09	1.71 ± 0.12	3.26 ± 0.28	2.73 ± 0.21	1.64 ± 0.12	1.74 ± 0.13	2.37 ± 0.18	0.22 ± 0.02	0.33 ± 0.03	6.62 ± 0.50	8.90 ± 0.58	2.29 ± 0.20	0.73 ± 0.06	0.58 ± 0.04	0.41 ± 0.03
R35	1.63 ± 0.10	1.44 ± 0.09	3.11 ± 0.27	3.60 ± 0.27	1.01 ± 0.07	1.81 ± 0.12	2.22 ± 0.17	0.26 ± 0.02	0.34 ± 0.03	7.09 ± 0.53	7.07 ± 0.52	2.49 ± 0.20	1.61 ± 0.13	1.04 ± 0.08	0.75 ± 0.06
R36	1.64 ± 0.10	2.55 ± 0.19	3.90 ± 0.33	3.30 ± 0.25	0.81 ± 0.06	2.33 ± 0.16	0.88 ± 0.06	0.34 ± 0.02	0.64 ± 0.04	6.25 ± 0.41	4.32 ± 0.28	2.30 ± 0.18	1.35 ± 0.09	0.66 ± 0.05	0.40 ± 0.03
R37	Nd	3.14 ± 0.22	1.87 ± 0.14	3.06 ± 0.18	0.64 ± 0.05	1.35 ± 0.08	3.68 ± 0.25	0.82 ± 0.05	0.26 ± 0.02	6.07 ± 0.44	7.48 ± 0.54	2.47 ± 0.20	0.85 ± 0.05	0.80 ± 0.05	1.34 ± 0.08
R38	3.78 ± 0.28	1.42 ± 0.09	6.14 ± 0.42	5.32 ± 0.40	3.60 ± 0.29	3.96 ± 0.34	3.74 ± 0.28	1.09 ± 0.07	0.76 ± 0.05	13.10 ± 0.99	17.70 ± 1.21	4.08 ± 0.27	1.68 ± 0.11	1.45 ± 0.09	0.93 ± 0.06
R39	1.05 ±	4.51 ±	1.38 ±	3.62 ±	1.07 ±	2.08 ±	4.36 ±	0.52 ±	0.44 ±	11.28 ±	11.82 ±	4.12 ±	1.44 ±	0.98 ±	0.82 ±

	0.07	0.32	0.11	0.19	0.07	0.14	0.26	0.04	0.03	0.80	0.84	0.25	0.09	0.06	0.05
R40	0.78 ± 0.05	1.22 ± 0.07	1.20 ± 0.06	1.65 ± 0.08	2.25 ± 0.13	1.14 ± 0.07	2.27 ± 0.14	0.09 ± 0.01	0.21 ± 0.02	4.19 ± 0.25	10.40 ± 0.62	2.33 ± 0.16	0.95 ± 0.06	0.73 ± 0.05	0.50 ± 0.03
R41	0.89 ± 0.06	1.37 ± 0.08	1.16 ± 0.07	1.78 ± 0.09	0.93 ± 0.05	1.06 ± 0.06	2.08 ± 0.13	0.10 ± 0.01	0.21 ± 0.02	4.40 ± 0.26	8.24 ± 0.50	2.68 ± 0.17	2.21 ± 0.14	1.40 ± 0.09	1.04 ± 0.06
R42	1.27 ± 0.09	1.46 ± 0.10	3.48 ± 0.26	3.90 ± 0.29	0.79 ± 0.05	1.95 ± 0.12	0.93 ± 0.06	0.27 ± 0.02	0.42 ± 0.03	4.85 ± 0.31	4.75 ± 0.31	2.28 ± 0.15	1.25 ± 0.08	0.70 ± 0.04	0.45 ± 0.03
R43	1.69 ± 0.11	2.43 ± 0.18	4.22 ± 0.34	0.80 ± 0.06	1.69 ± 0.12	0.54 ± 0.04	0.63 ± 0.04	0.03 ± 0.01	0.09 ± 0.01	2.06 ± 0.13	2.02 ± 0.12	2.34 ± 0.16	3.16 ± 0.20	1.89 ± 0.11	0.52 ± 0.04

Supplement Table S4. Method validation parameters for the compounds identified in five types of honey, including linearity, R^2, LOD, LOQ, recovery, and precision (RSD%).

Compound	Linearity (mg/L)	R^2	LOD (mg/L)	LOQ (mg/L)	Recovery (%)	Precision (RSD%)	Calibration curves
A1	0.44–65	0.998	0.025	0.044	95–103	3.31	y=16795x-120167
A2	0.96–153	0.998	0.094	0.105	93–107	2.2	y=14965x-137890
A3	0.76–116	0.998	0.083	0.275	92–104	1.56	y=2779x-15395
A4	0.64–68	0.995	0.067	0.095	95–106	3.11	y=2027x-18898
A5	0.24–124	0.996	0.088	0.069	95–102	1.94	y=1685x-40836
A6	0.24–55	0.995	0.082	0.162	92–105	3.85	y=1713.8x-158981
A7	0.15–186	0.998	0.027	0.296	93–102	3.88	y=7566.07x-199483
A8	0.88–89	0.997	0.09	0.095	92–105	3.79	y=3016.1x+92827
B1	0.64–149	0.996	0.059	0.211	97–102	2.43	y=1487x-55623
B2	0.74–97	0.995	0.083	0.236	95–107	1.54	y=6485.5x-40808
B3	0.12–128	0.996	0.091	0.094	96–106	3.82	y=3345.7x-188450
B4	0.97–132	0.996	0.039	0.227	96–105	2.57	y=4383.2x-50000
B5	0.85–78	0.998	0.02	0.129	94–104	3.92	y=4581x-274502
B6	0.29–195	0.998	0.031	0.201	92–102	3.91	y=4625.4x-11517
B7	0.26–166	0.999	0.048	0.201	92–102	3.63	y=1760.3x-35143
B8	0.27–191	0.997	0.084	0.175	94–105	2.24	y=4545.1x- 10916
B9	0.37–184	0.995	0.087	0.054	94–104	2.46	y=7793.9x -18443
C1	0.57–140	0.998	0.011	0.256	94–104	3.63	y=1878x-96367
C2	0.49–188	0.998	0.056	0.117	95–107	2.29	y=3264x-117073
C3	0.36–63	0.997	0.048	0.08	97–107	1.92	y=3264x-117073
C4	0.65–79	0.998	0.03	0.041	92–107	2.89	y=3264x-117073
C5	0.23–57	0.997	0.021	0.19	95–107	3.84	y=3264x-117073
C6	0.36–99	0.997	0.04	0.213	94–107	3.24	y=1878x-96367
C7	0.43–108	0.997	0.095	0.034	92–107	2.93	y=1878x-96367
C8	0.51–91	0.995	0.039	0.168	95–106	1.74	y=1878x-96367

C9	0.81–174	0.995	0.057	0.091	97–104	3.04	y=1878x-96367
C10	0.28–104	0.995	0.073	0.204	95–107	3.98	y=1869x-97664
D1	0.56–92	0.998	0.043	0.077	97–104	1.85	y=1750x-16020
D2	0.63–131	0.996	0.097	0.217	94–104	2.8	y=1695.8x-498677
D3	0.14–71	0.997	0.097	0.134	97–103	3.69	y=2795x-466413
D4	0.65–170	0.999	0.033	0.283	92–106	3.35	y=7794.2x-18454
D5	0.25–61	0.996	0.055	0.067	94–107	3.24	y=2708x-1076590
E1	0.16–198	0.997	0.037	0.122	92–102	3.26	y=526.14x-111770
E2	0.95–166	0.998	0.036	0.061	96–105	2.4	y=4513x+135049
E3	0.97–80	0.996	0.013	0.28	93–102	2.23	y=4396x+176015
E4	0.83–51	0.995	0.065	0.267	97–106	3.52	y=879x-70024
F1	0.37–172	0.996	0.055	0.1	93–105	3.53	y=2699.8x-901946

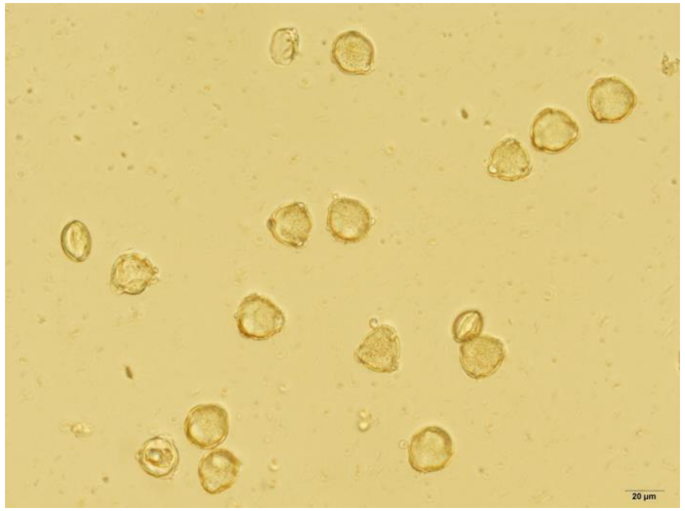

Loquat honey

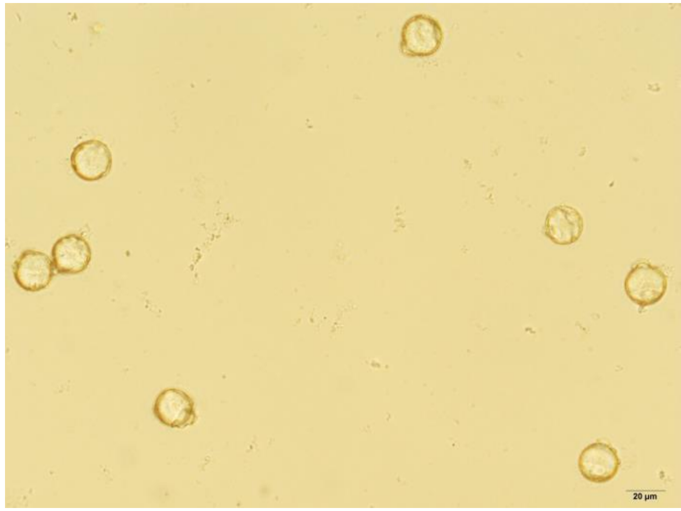

Pomegranate honey

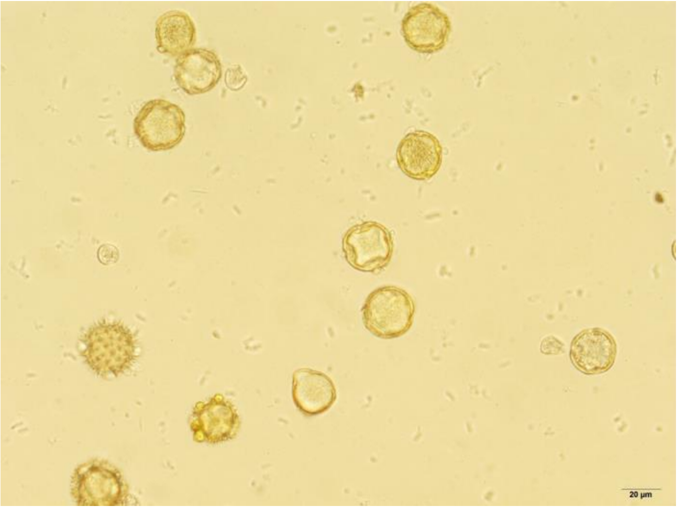

Citrus honey

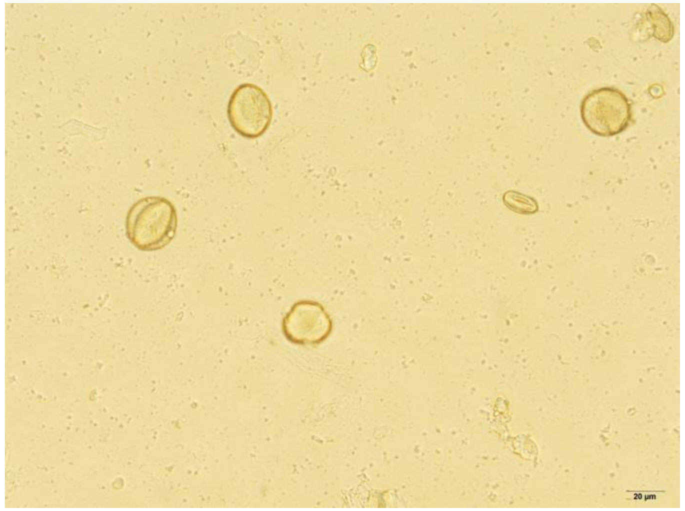

Apple honey

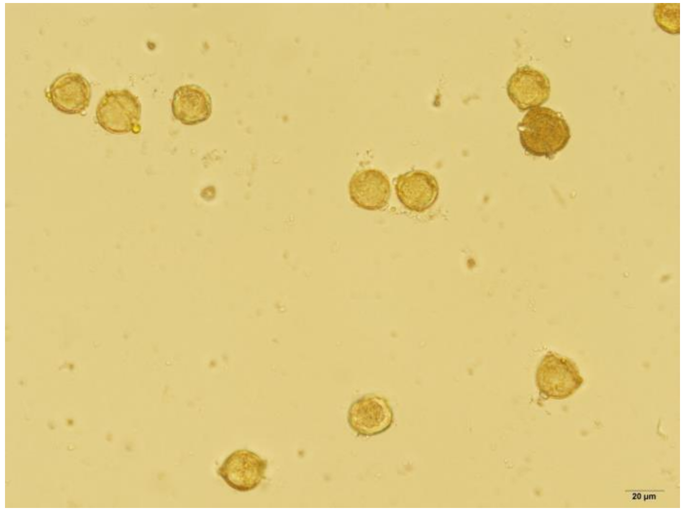

Blueberry honey

Supplementary Figure S1. Pollen grain photomicrographs of five types of honey.

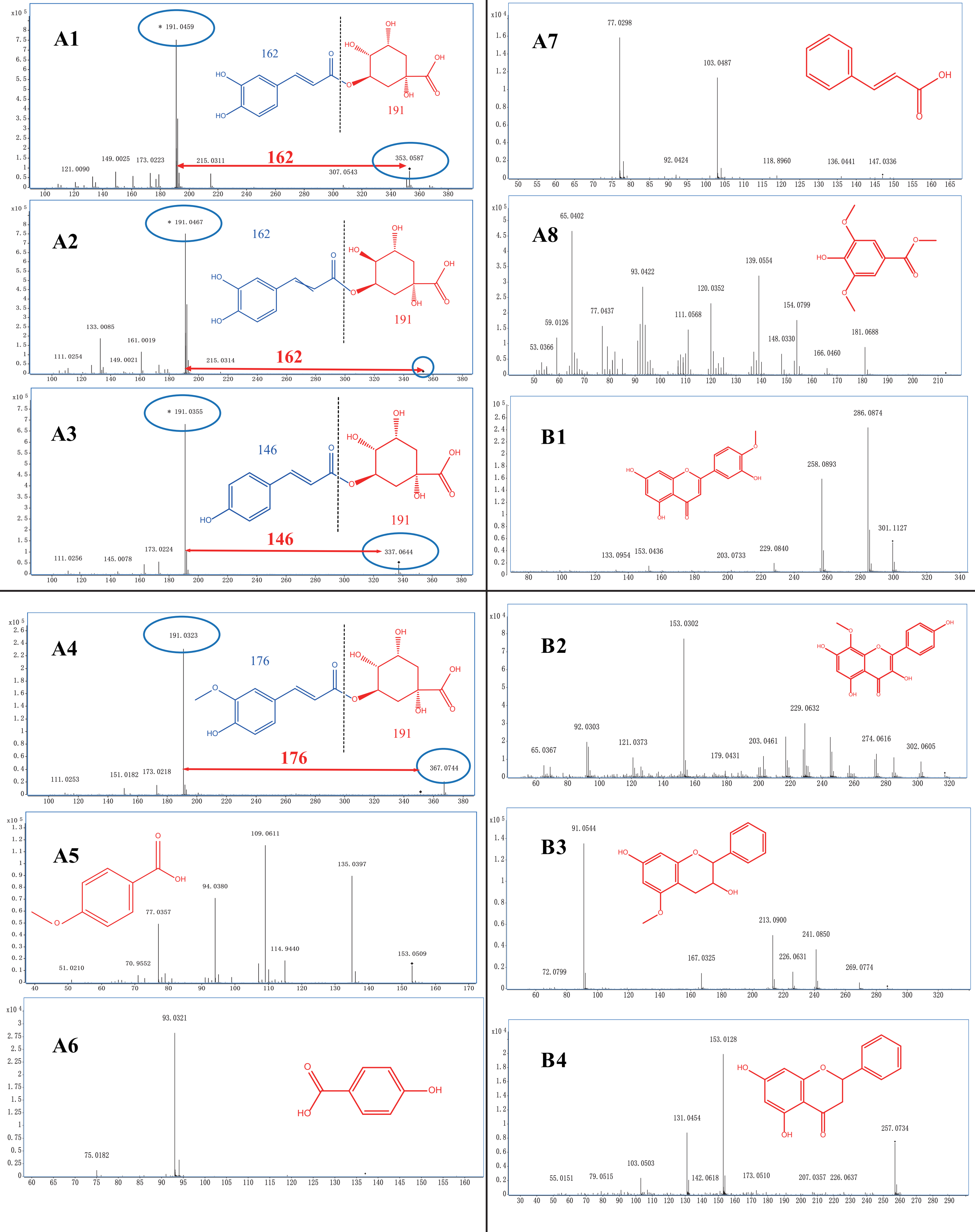

A1
* 191.0459
162
191
121.0090
149.0025
173.0223
215.0311
307.0543
353.0587
A2
* 191.0467
162
191
111.0254
133.0085
149.0021
161.0019
215.0314
A3
* 191.0355
146
191
111.0256
145.0078
173.0224
337.0644
A4
191.0323
176
191
111.0253
151.0182
173.0218
367.0744
A5
51.0210
70.9552
77.0357
94.0380
109.0611
114.9440
135.0397
153.0509
A6
75.0182
93.0321
A7
77.0298
92.0424
103.0487
118.8960
136.0441
147.0336
A8
53.0366
59.0126
65.0402
77.0437
93.0422
111.0568
120.0352
139.0554
148.0330
154.0799
166.0460
181.0688
B1
133.0954
153.0436
203.0733
229.0840
258.0893
286.0874
301.1127
B2
65.0367
92.0303
121.0373
153.0302
179.0431
203.0461
229.0632
274.0616
302.0605
B3
72.0799
91.0544
167.0325
213.0900
226.0631
241.0850
269.0774
B4
55.0151
79.0515
103.0503
131.0454
142.0618
153.0128
173.0510
207.0357
226.0637
257.0734

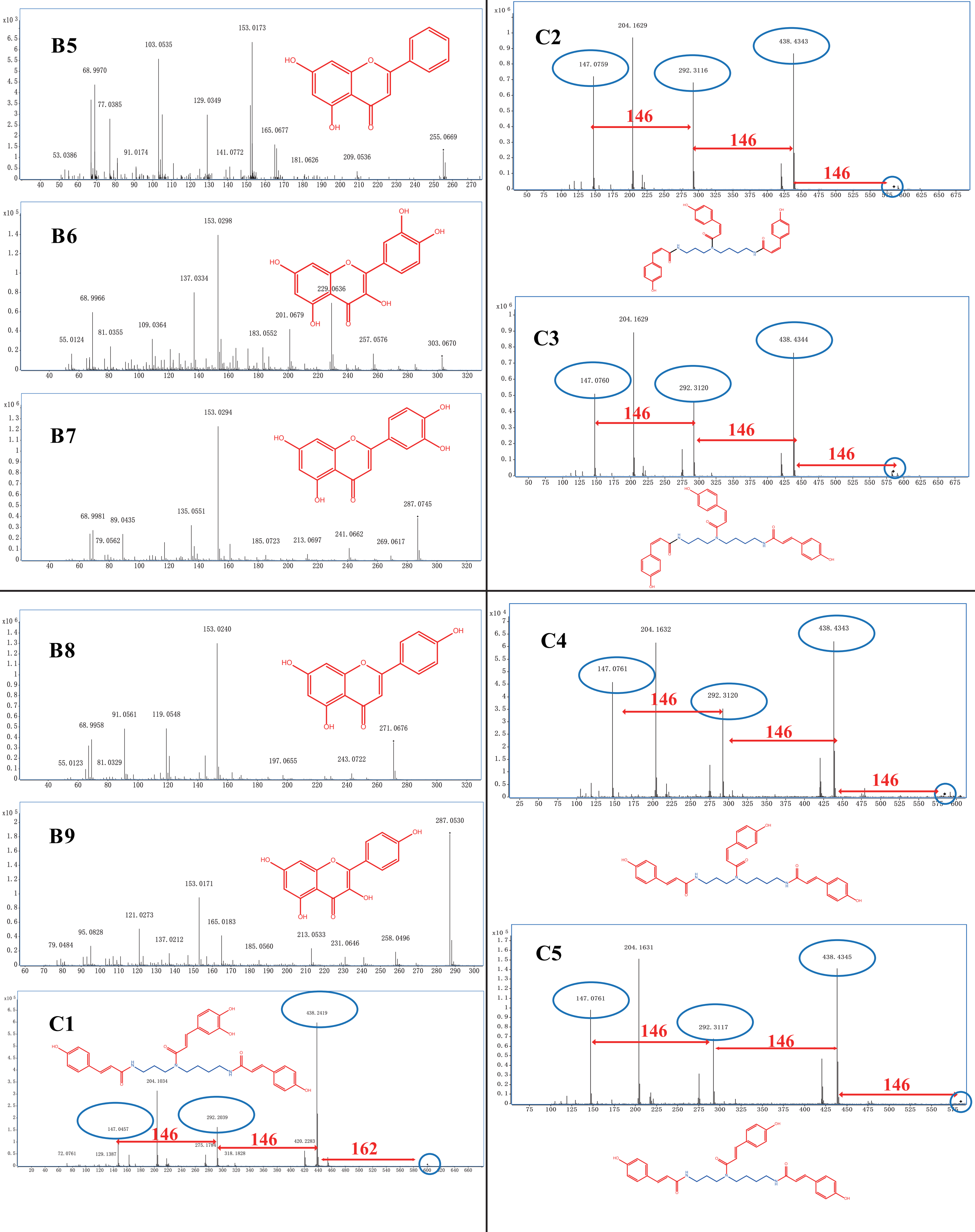

B5
x10 3
53. 0386
68. 9970
77. 0385
91. 0174
103. 0535
129. 0349
141. 0772
153. 0173
165. 0677
181. 0626
209. 0536
255. 0669
HO
OH
O
B6
x10 5
55. 0124
68. 9966
81. 0355
109. 0364
137. 0334
153. 0298
183. 0552
201. 0679
229. 0636
257. 0576
303. 0670
OH
HO
B7
x10 6
68. 9981
79. 0562
89. 0435
135. 0551
153. 0294
185. 0723
213. 0697
241. 0662
269. 0617
287. 0745
B8
x10 6
55. 0123
68. 9958
81. 0329
91. 0561
119. 0548
153. 0240
197. 0655
243. 0722
271. 0676
B9
x10 5
79. 0484
95. 0828
121. 0273
137. 0212
153. 0171
165. 0183
185. 0560
213. 0533
231. 0646
258. 0496
287. 0530
C1
x10 5
438. 2419
204. 1034
147. 0457
292. 2039
72. 0761
129. 1387
275. 1784
318. 1828
420. 2283
146
146
162
C2
x10 6
204. 1629
147. 0759
292. 3116
438. 4343
146
146
146
C3
x10 6
204. 1629
438. 4344
147. 0760
292. 3120
146
146
146
C4
x10 4
204. 1632
438. 4343
147. 0761
292. 3120
146
146
146
C5
x10 5
204. 1631
438. 4345
147. 0761
292. 3117
146
146
146

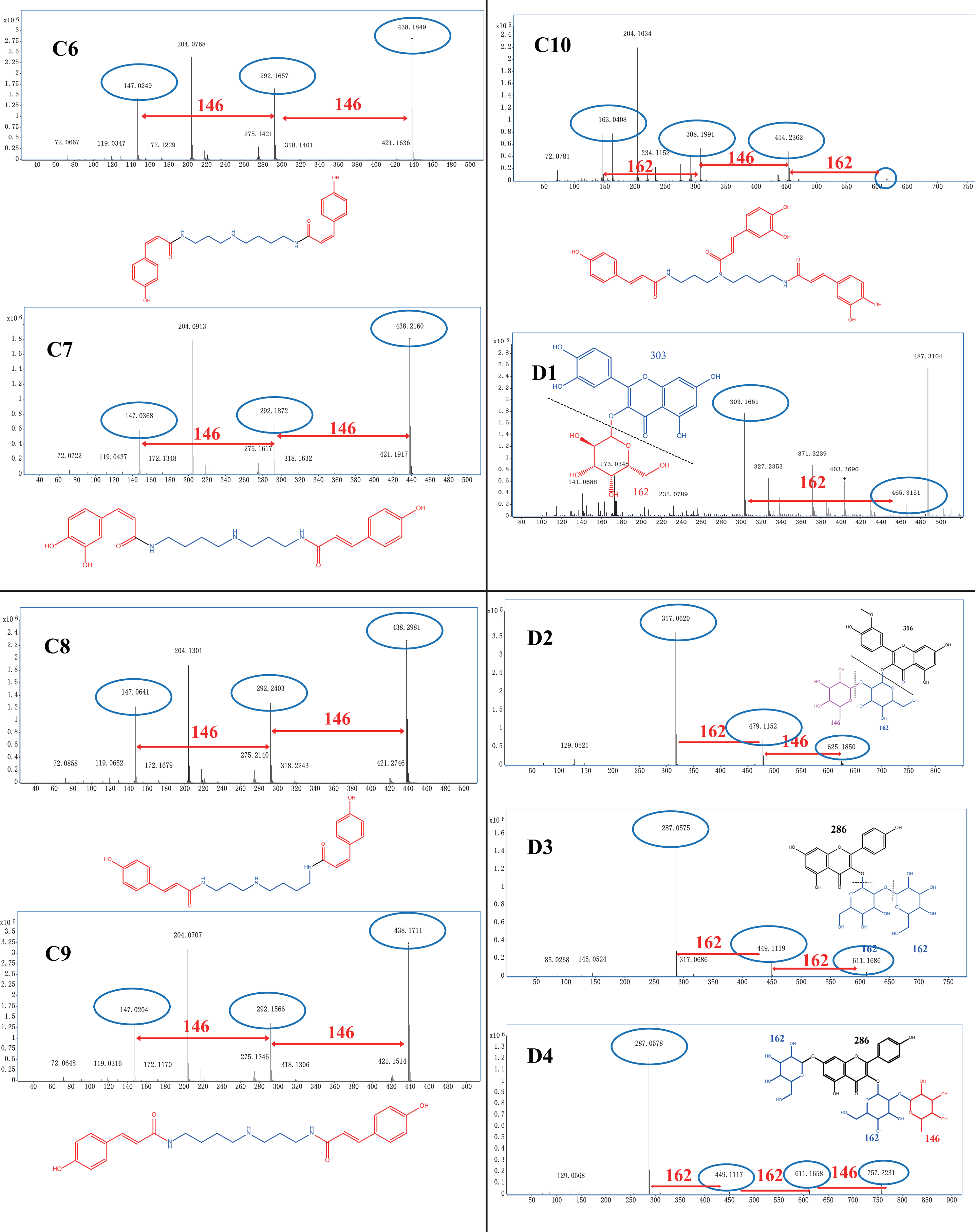

C6
x10 6
72. 0667
119. 0347
147. 0249
172. 1229
204. 0768
275. 1421
292. 1657
318. 1401
421. 1636
438. 1849
146
146
OH
O
N H
N H
N H
OH
C10
x10 5
72. 0781
163. 0408
204. 1034
234. 1152
308. 1991
454. 2362
162
146
162
HO
OH
OH
OH
OH
O
O
O
N
H
N H
C7
x10 6
72. 0722
119. 0437
147. 0368
172. 1348
204. 0913
275. 1617
292. 1872
318. 1632
421. 1917
438. 2160
146
146
HO
OH
O
N H
H N
H N
O
OH
D1
x10 5
303
HO
HO
O
OH
OH
O
O
HO
HO
OH
OH
173. 0345
141. 0688
162
232. 0789
303. 1661
327. 2353
371. 3239
403. 3690
465. 3151
487. 3104
C8
x10 6
72. 0858
119. 0652
147. 0641
172. 1679
204. 1301
275. 2140
292. 2403
318. 2243
421. 2746
438. 2981
146
146
HO
OH
HN
N H
N H
O
O
C9
x10 6
72. 0648
119. 0316
147. 0204
172. 1170
204. 0707
275. 1346
292. 1566
318. 1306
421. 1514
438. 1711
146
146
HO
OH
O
N H
H N
H N
O
D2
x10 5
129. 0521
317. 0620
479. 1152
625. 1850
162
146
316
146
162
D3
x10 6
85. 0268
145. 0524
287. 0575
317. 0686
449. 1119
611. 1686
162
162
286
162
162
D4
x10 6
129. 0568
287. 0578
449. 1117
611. 1658
757. 2231
162
162
146
286
162
162
146

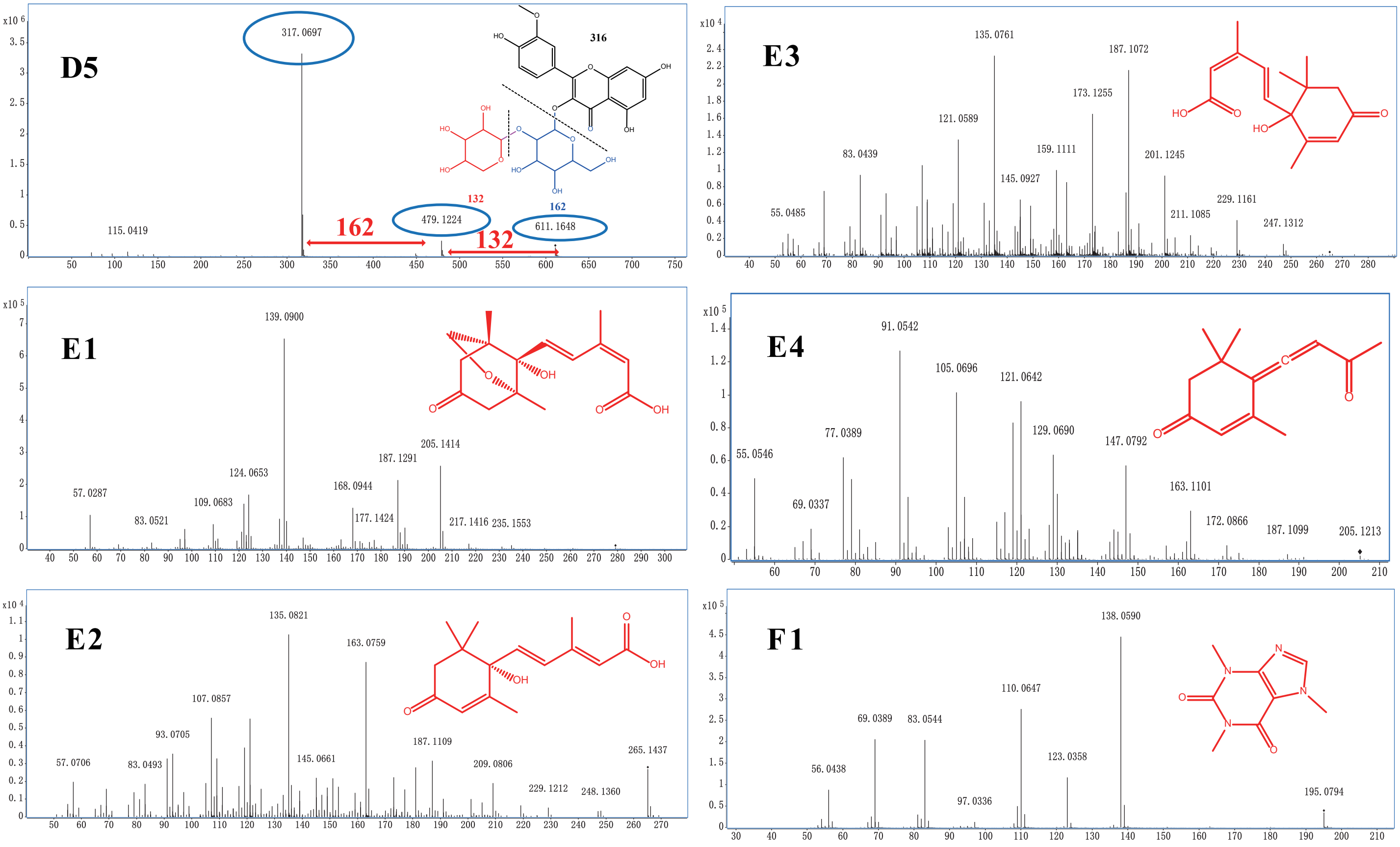

Supplementary Figure S2. MS/MS spectra (ESI) and fragment cleavage patterns of phytochemicals

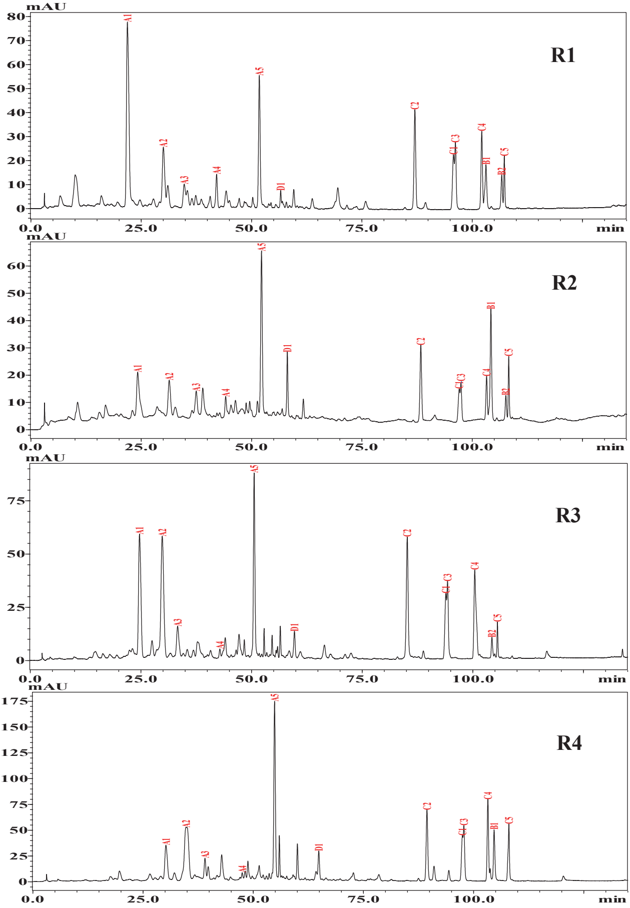

mAU
R1
A1
A2
A3
A4
A5
D1
C2
C1
C3
C4
B1
B2
C5
0.0
25.0
50.0
75.0
100.0
min
R2
R3
R4

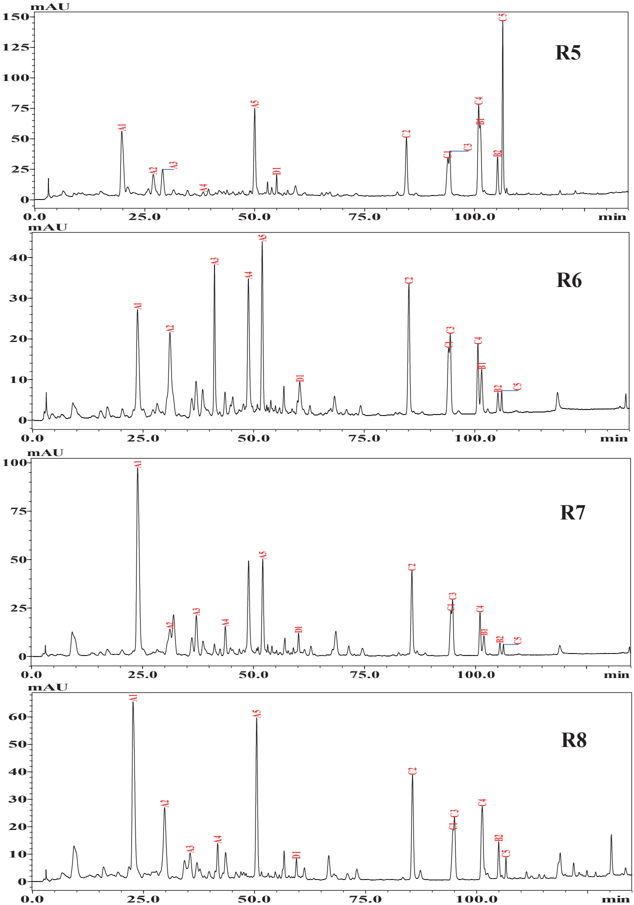

R5
mAU
min
A1
A2
A3
A4
A5
D1
C2
C1
C3
C4
B1
B2
C5
0
25
50
75
100
125
150
0.0
25.0
50.0
75.0
100.0
R6
mAU
min
0
10
20
30
40
R7
mAU
min
0
25
50
75
100
R8
mAU
min
0
10
20
30
40
50
60

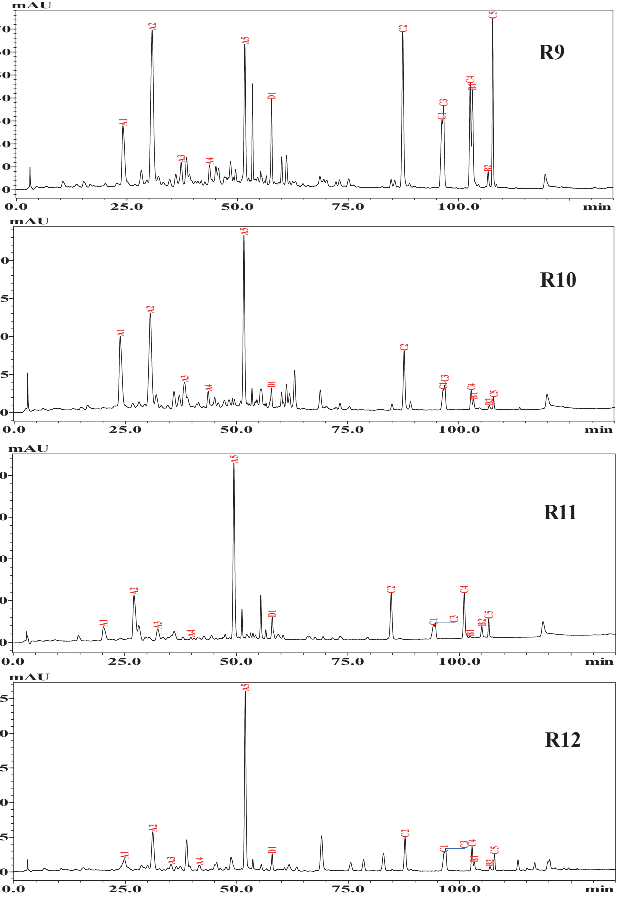
mAU
R9
0.0
25.0
50.0
75.0
100.0
min
A1
A2
A3
A4
A5
D1
C2
C1
C3
C4
B1
B2
C5
R10
R11
R12

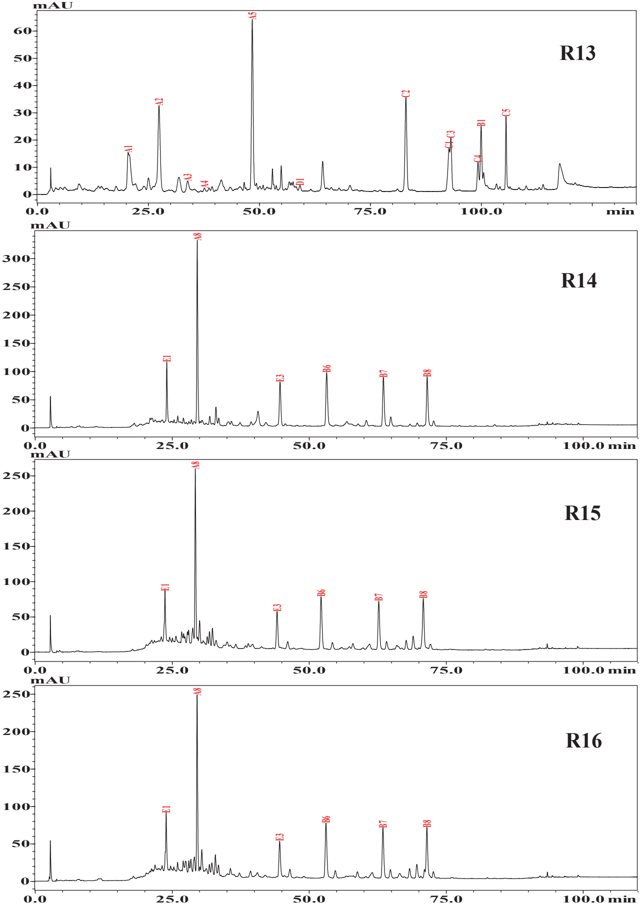

mAU
R13
A1
A2
A3
A4
A5
D1
C2
C1
C3
C4
B1
C5
min
mAU
R14
E1
A8
E3
B6
B7
B8
min
mAU
R15
E1
A8
E3
B6
B7
B8
min
mAU
R16
E1
A8
E3
B6
B7
B8
min

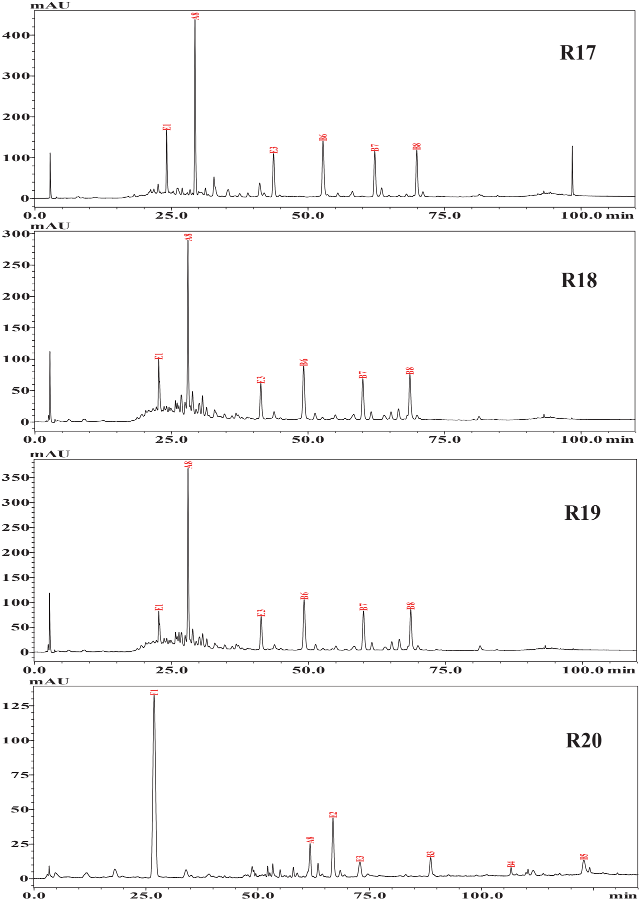
mAU
R17
E1
A8
E3
B6
B7
B8
0.0
25.0
50.0
75.0
100.0 min
mAU
R18
E1
A8
E3
B6
B7
B8
0.0
25.0
50.0
75.0
100.0 min
mAU
R19
E1
A8
E3
B6
B7
B8
0.0
25.0
50.0
75.0
100.0 min
mAU
R20
F1
A8
E2
E3
B3
B4
B5
0.0
25.0
50.0
75.0
100.0
min

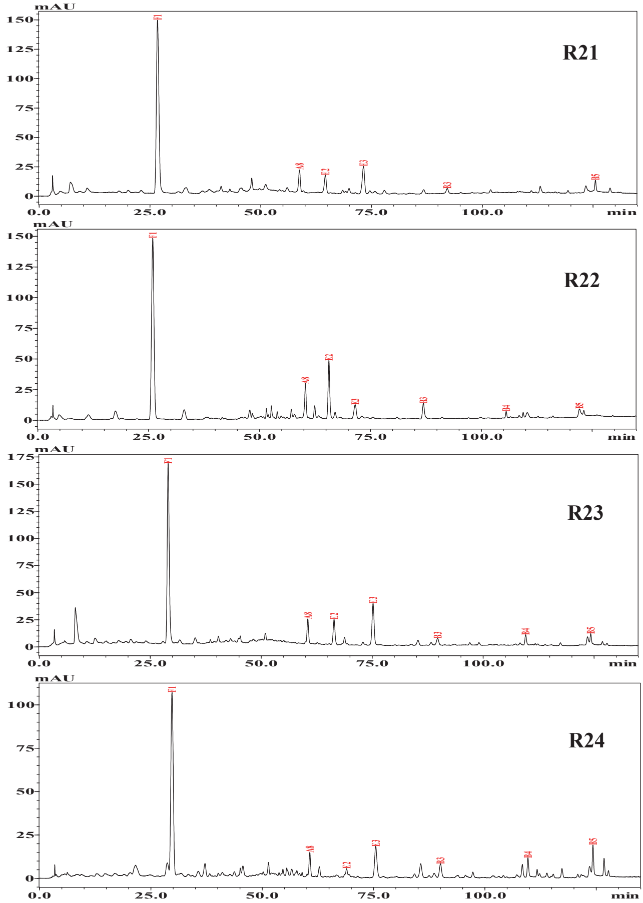

R21
R22
R23
R24
mAU
min
F1
A8
E2
E3
B3
B4
B5
0.0
25.0
50.0
75.0
100.0

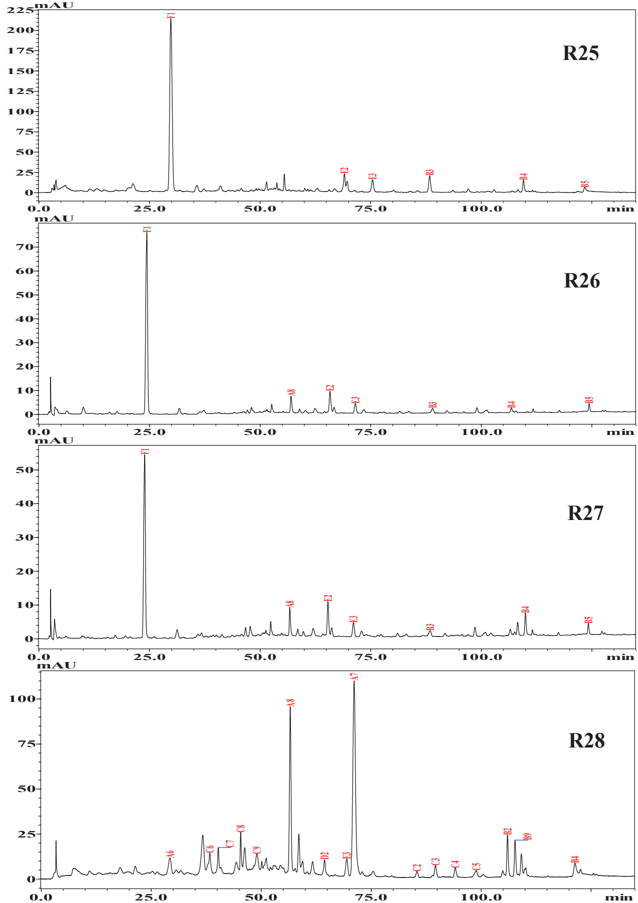

R25
R26
R27
R28
mAU
min
F1
E2
E3
B3
B4
B5
A8
A6
C6
C7
C8
C9
D2
A7
C2
C3
C4
C5
B2
B9

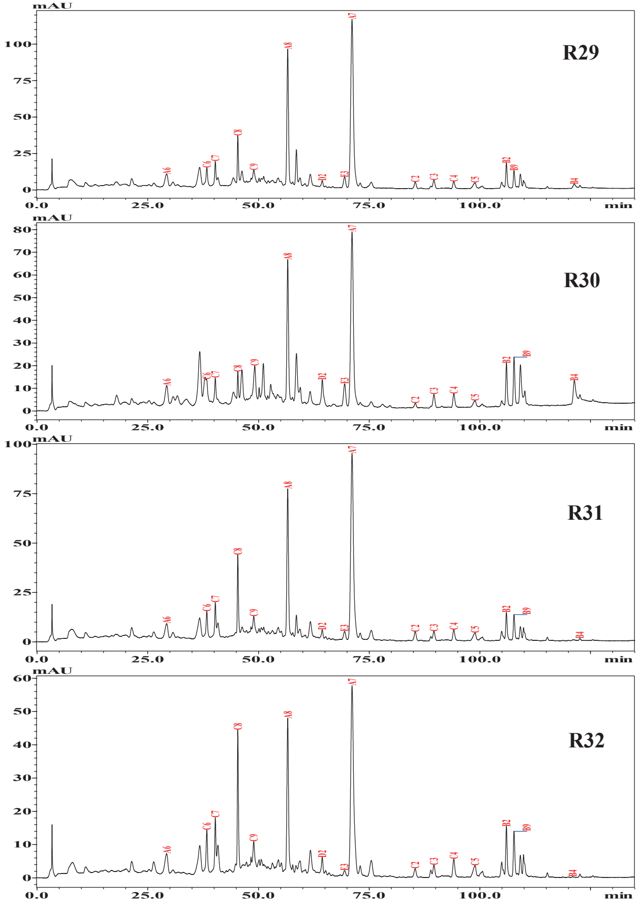

R29
mAU
100
75
50
25
0
A6
C6
C7
C8
C9
A8
D2
E3
A7
C2
C3
C4
C5
B2
B9
B4
0.0
25.0
50.0
75.0
100.0
min
R30
mAU
80
70
60
50
40
30
20
10
0
R31
mAU
100
75
50
25
0
R32
mAU
60
50
40
30
20
10
0

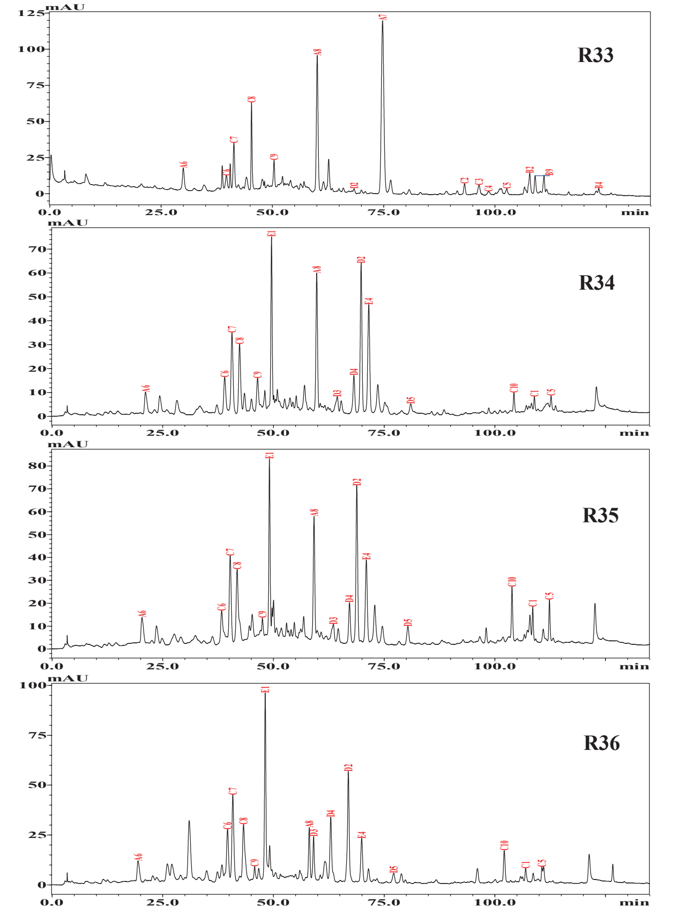

R33
mAU
125
100
75
50
25
0
0.0
25.0
50.0
75.0
100.0
min
A6
C6
C7
C8
C9
A8
D2
A7
C2
C3
C4
C5
B2
B9
B4
R34
mAU
70
60
50
40
30
20
10
0
A6
C6
C7
C8
C9
E1
A8
D3
D4
D2
E4
D5
C10
C1
C5
R35
mAU
80
70
60
50
40
30
20
10
0
A6
C6
C7
C8
C9
E1
A8
D3
D4
D2
E4
D5
C10
C1
C5
R36
mAU
100
75
50
25
0
A6
C6
C7
C8
C9
E1
A8
D3
D4
D2
E4
D5
C10
C1
C5

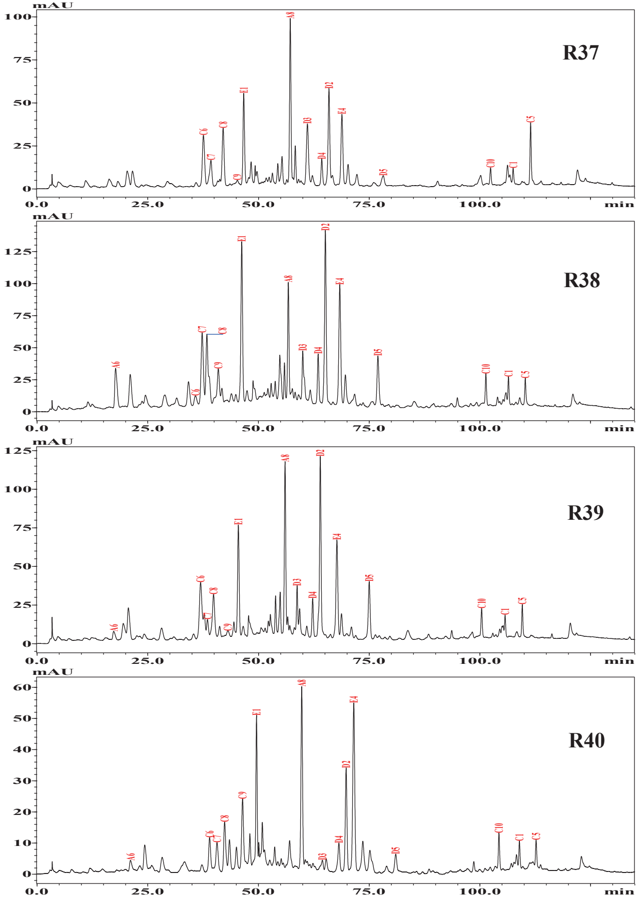
mAU
R37
A8
E1
D2
E4
D3
C6
C8
C7
D4
C9
D5
C10
C1
C5
min
0.0
25.0
50.0
75.0
100.0
R38
A6
C7
C8
C6
C9
E1
A8
D3
D4
D2
E4
D5
C10
C1
C5
R39
A6
C6
C7
C8
C9
E1
A8
D3
D4
D2
E4
D5
C10
C1
C5
R40
A6
C6
C7
C8
C9
E1
A8
D3
D4
D2
E4
D5
C10
C1
C5

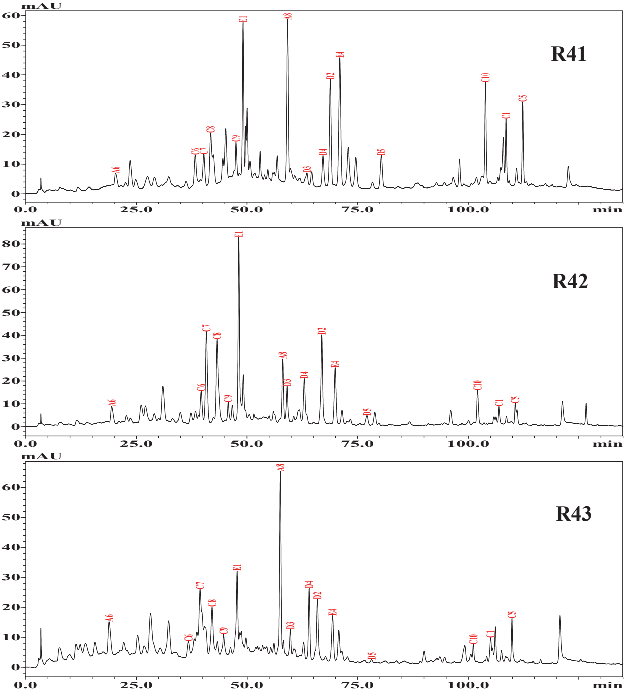

Supplementary Figure S3. HPLC profiles of five types of raw honey.

Note: R1 to R13 represent loquat honey sample 1 to 13； R14 to R19 represent pomegranate honey sample 1 to 6； R20 to R27 represent citrus honey sample 1 to 8； R28 to R33 represent apple honey sample 1 to 6； R34 to R43 represent blueberry honey sample 1 to 10.

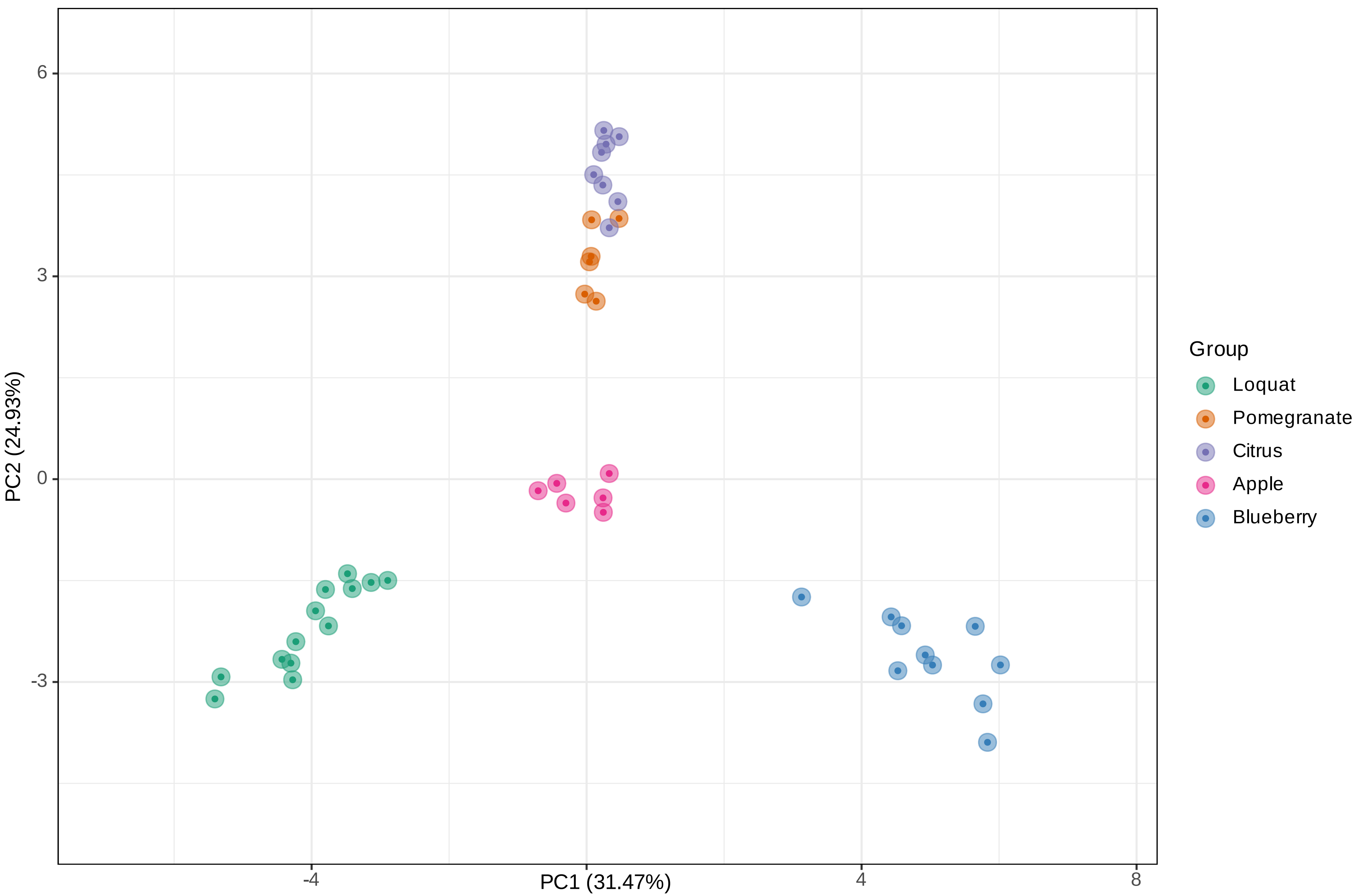

Supplementary Figure S4. PCA score plot showing the clustering of five monofloral honey samples according to their phytochemical profiles.
